# Supplementary material for: A qualitative study to investigate pharmacovigilance systems in Dubai hospitals
Source: PLoS One. 2025 Sep 10;20(9):e0331929. doi: 10.1371/journal.pone.0331929 (PMC12422479; doi:10.1371/journal.pone.0331929)
Supplement: S1 File — (ZIP) [file pone.0331929.s001.zip › M1.docx]

Speaker 1: Okay. Okay. Good morning Dr. Khan. How are you?

Speaker 2: Good, good. Thank you.

Speaker 1: Today I'll do with you an interview about the pharmacovigilance and ADR reporting in ,,,,,,,Hospital. Can you please introduce yourself?

Speaker 2: Yeah, my name is ,,,,,,,. I graduated as a doctor of Pharmacy. Then I did master in Clinical Pharmacy and master in business administration. I'm working in UA E around 12 years in the private sector and right now I'm working in ,,,,,,, hospital as a pharmacy manager.

Speaker 1: Okay, excellent. And here in ,,,,,,, hospital for how many Years?

Speaker 2: Two years. Two years. So I joined in September, 2020.

Speaker 1: Okay. And before you

Speaker 2: Were I was working in medical clinic hospital in Abu Dhabi. Okay,

Speaker 1: Nice. So you collect experiences From different places in the country?

Speaker 2: Yeah, so I've been in Mediclinic so they're also 10 years. I was so closely working with clinical team and JCIA and quality. So I had the experience with me, medication error and ADR, adverse drug reactions.

Speaker 1: Okay. Okay. So you have as you said, experience with the ADR reporting both in here for Emirates hospital and the Mediclinic Abu Dhabi. So here you are the one who's responsible for ADRs reporting?

Speaker 2: Yes, so as I am the clinical pharmacist in the hospital, I'm the one responsible to analyze the ADRs, medication errors, near misses in coordination with the quality, quality and risk management department or QRM. So I categorize and then we have the KPI on this medication errors ADR and near misses, which we report on a monthly basis to our pharmacy and therapeutic committee meetings.

Speaker 1: Okay. Understand that. So can you tell me about the flow, the process of reporting the ADR starting from for outpatient pharmacies? Differently than inpatient?

Speaker 2: Yes, exactly. So first I would highlight you for the inpatient. Okay. I would like to tell you because we prepare the policies and procedure, we have one policy called reporting of monitoring of medication effects and reporting of adverse drug reaction as per JCI. So medication management and use standard is very important in any hospital we are JCI accredited and as per MMU chapter. So we need to have a proper pharmac process and monitoring of the medication effects. So for that we had the policy. So what happened? So once you administer the medication at the inpatient level or at the hospital level. So we need to monitor if nurses will monitor the effects of the medication. It includes either it is effective or not effective, like if you are giving painkiller. Also it includes if the patient develop any reaction or any side effects. So she need to monitor this one.

Speaker 2: So we have a timeline based on the injectables, orals and all that. So if she finds any allergic reaction or any anaphylaxis reaction to any medication, first thing she will stop the medication. She will report to the physician or the prescriber or the consultant physician. They will stabilize the patient. So if there is any allergic reaction or anaphylaxis. So they'll try to stabilize after that as per policy they need to report once the patient is stable. So they need to report it, whoever report it? No. So we have online portal, it's called OVR and medication error, what is called again OVR Portal O our internal portal online by default all the staff they have the username and password to access this. Whoever joined the key, I mean the healthcare professional, they will have an access. So they will go to the portal. There are some information requires they will fill and they will report it. So by submitting this A DR or MER medication error, it'll go to our quality and risk management department, Q-R-M-Q-R-M. They will review it and then they will assign to the concerned person

Speaker 1: In the quality what their qualification there in the quality address they are pharmacist?

Speaker 2: In the quality, there is a doctor, there is a nurses. Okay. So by default any medication errors related or adverse drug reaction related they will assign to clinical pharmacist. Okay. By default, I mean so I am the one being a clinical pharmacist assigned for this. So it'll come to me and to the director of pharmacy for sure. So I will review, I investigate, analyze, communicate with the concern department what happened and then we categorize this A DR er and if it is something like lethal or sentinel events, so we need to report it to DHA or MOH also. So there is online link also to reporting to MOH or DHA and this is one of our KPI clinical KPI, which we report to our pharmacy and therapeutic committee also. Okay. So we'll see if based on the analysis then we'll develop the action plans and then execute usually if we need to call any FMEA meeting. Okay. So then the team sits together, multidisciplinary team, they develop the action plans and execute it in order to avoid such reactions in the future.

Speaker 1: Okay, excellent. So as you said you developed action plan and you will do reporting for the DHA right Directly.

Speaker 2: Yes.

Speaker 1: Once you got the ADR Reports.

Speaker 2: So we need to report in addition to that quarterly reporting is being done by QRM to DHA on the medication error and the ADRs including All this.

Speaker 1: You are reporting to DHA or to MOH as they are linked to each other?

Speaker 2: So once it is open it's also connected to MOH. So it goes to them also.

Speaker 1: And you are reporting as you said, every quarter?

Speaker 2: Yeah, it is. I report to quality and then quality they report it to them.

Speaker 1: Okay, so the quality, they Report quarterly?

Speaker 2: Yes.

Speaker 1: So beside that you are reporting for them directly. Once it's happened they also submit another report that what you mean the quality?

Speaker 2: So the cycle is like this: Any staff, they fill the form online form, it goes to quality from quality to clinical pharmacists for analysis, review, feedback, action plan, then go back to quality if any meeting is required will be conducted for the execution of any action plan and the quality on a quarterly basis report to DHA and MH.

Speaker 1: Okay, so it is not like directly that you have to report to them?

Speaker 2: No, internally I will report it so to the quality and quality is the one.

Speaker 1: Even if it is like a serious side.

Speaker 2: If serious or sentinel, this is they say within 15 days MOH stays need to be but until now we don't have any sentinel or serious this one. But DHA they requires to be reported within 15 days any sentinel.

Speaker 1: Okay. And what about the outpatient, what is the process?

Speaker 2: So the outpatient also if patient reported from home that he took this medication and he develop allergic reaction for sure he'll report it to the hospital. Okay. They call the pharmacist,

Speaker 2: They told they call the pharmacy to the pharmacy or to the doctor. So then again it'll be escalated to the quality. Quality will report so to the clinical pharmacist analysis will happen and all that. Yeah. And now we have one inpatient also have that

Speaker 1: This is when it happened?

Speaker 2: It happened three, four months back, three four months. So the patient might be allergic to Bruen or Ving. So it's like a class of answers. So he was prescribed another answers. So considering doctor and also pharmacist.

Speaker 1: So you analyzed the case?

Speaker 2: Yeah, we analyzed, patient complained that he had the suffocation after taking this medication. So then we analyze and we found that this is a cross sensitivity from one answer to another one.

Speaker 1: Okay. The patient, he was reported to the physician or to the pharmacy?

Speaker 2: He reported to the physician. Physician and our patient experience, we have patient experience department also.

Speaker 1: What is the patient experience Department?

Speaker 2: If patient have any issue with regards to the hospital or any surveys so they can report also.

Speaker 1: Okay. Directly To patient experience?

Speaker 2: Yes, So then patient experience department execute or direct to the concern department from where he has that issue or concern.

Speaker 1: Nice. Okay. So how many the frequency now you said that during you are here have been two years. So if we can say the frequency of ADR reporting.

Speaker 2: See we monitor monthly basis. Okay. But the frequency of ADR is very low but the MER medication error reporting is more because pharmacy also actively participate in this and we do the intervention review. But the A DR you can say is very less so might be you can say due to it's not related allergic reactions are more so not about the side effects of the medications and all that. So I would say a DR reporting is very minimal or less as compared to MER.

Speaker 1: Okay. And why the ME is more?

Speaker 2: So we can say we are a new hospital system is being developed so it's automated and physicians are new, physicians are joining so they need the training over the prescribings and all these issues. So you can say our benchmark was like a hundred percent should be near means it should be prevented before reaching to the patient. So we are like 99% we are preventing before it reaches to the patient. So very rare it goes to the patient like maybe one or two per month and also like it is a category C, it did not cause any harm till now we don't have any error which caused the harm to the patient.

Speaker 1: And again who discovered this medication errors?

Speaker 2: It can happen at any level. See the medication error we see it can happen at prescribing level, dispensing level and administration level. Okay. So mainly if you say due to the active participation of pharmacists, we stop it at the level of dispensing.

Speaker 1: Okay, excellent.

Speaker 2: So clinical pharmacist or the pharmacist, they review the prescription appropriateness and they will stop it.

Speaker 1: And this happened for the inpatient and outpatient?

Speaker 2: Both. Both. Both. So we had for both and we have a mechanism to document this. We have in our C-P-O-A-U or HIS we have called pharmacy intervention system. So when they review pharmacist document in the SAE, which is our HAS, what was the intervention done? So it could be either wrong dose, wrong route of administration or mainly these are the concerns under those overdose or wrong duration maybe

Speaker 1: Drug drug interaction.

Speaker 2: Drug drug interactions are there.

Speaker 1: Okay. And this is the job for the clinical pharmacist, right?

Speaker 2: We train all our pharmacists to be clinical pharmacists. So all pharmacists play this role.

Speaker 1: Okay. This is when they make the round, which is on the daily basis?

Speaker 2: make the round make when they're reviewing the orders, I mean when they receive a prescription, when they review they can also do this activity at that level and everything through the system.

Speaker 1: So nothing is manual here?

Speaker 2: No, just through the system. But for reviewing or doing verification, yes we do the telephonic conversation but documentation or reporting everything through online system.

Speaker 1: And what is the name of the ADR reporting system you have?

Speaker 2: We call it OVR. We have our own OVR portal which is further divided into if you have any OVR and then another tab is for MER medication error reporting and then sub tab for MER is a DR adverse drug reaction reporting.

Speaker 1: Okay, very nice. So did you take any training courses before for ADR reporting?

Speaker 2: Yes. I did, 4 years ago

Speaker 1: Did you take it at your working place or specified course?

Speaker 2: There are some online courses I did them, but also during our pharm D we were trained on this pharmacovigilance during my PMD and the masters.

Speaker 1: So this has pharm D you took it to from where?

Speaker 2: From Pakistan.

Speaker 1: Pakistan. So there you study this?

Speaker 2: Yeah, as the curriculum and also during the JCI surveys when we are getting prepared. So we are trained on this

Speaker 1: From the hospital.

Speaker 2: From the hospital here itself because wherever you work as a hospital pharmacist, so usually medication error monitoring is one of the KPI of the pharmacy department.

Speaker 1: And they give the training for all pharmacists in the Pharmacy?

Speaker 2: Yes, exactly. Yes because it's a part of KPI. So all the pharmacists they need to be aware. Okay, excellent. And also we run the knowledge fairs to educate not only the pharmacist, also to the nurses, to the physician about the importance of pharmacovigilance about the reporting of medication error or the ADRs.

Speaker 1: And this is arranged by external?

Speaker 2: You can say by the quality department in coordination with the pharmacy department.

Speaker 1: Okay and you arrange it for the nurses?

Speaker 2: Yes, nurses and physician.

Speaker 1: Excellent. Okay. So it is obligatory here we can say that reporting of ADR is obligatory.

Speaker 2: Obligatory in the hospital for everyone, the nurses, the patient, pharmacist and everyone whoever monitor.

Speaker 2: If they don't report it, then later on if we find it has not been a reported, so might be raise an OVR and I mean education will be done if it's repeated again and again. So might be a disciplinary.

Speaker 1: So this is like a policy for this one if you don't report it, there would be some steps have to be taken.

Speaker 2: Yes, exactly, exactly.

Speaker 1: Yeah.

Speaker 2: Okay. Because this is a policy mandatory, so if they don't report any allergic reaction, we have to mandatory report it. I mean there is no other way this is as a part of policy

Speaker 1: And after that what you would do and for example you discovered the case that the patient is allergic from this medication.

Speaker 2: So see there are two type of areas. One is idiosyncratic like medication, which we know that it could be happen like I will give you an example, beta lactam antibiotics or some patients are NSAIDs so we will check if it is in the system this patient was flagged for this allergy because one of the mandatory parameter when you are reviewing any medication orders to see if the patient has an allergy or no. So usually you will find no known medication allergy. So if this patient was not previously flagged or documented that there is no allergy reaction. So if we develop an allergy reaction to any medicine so it'll be documented in the system so that in the future we know that this patient is allergic to this and he will not get this medication Again some reactions are like anaphylaxis or which are dose related.

Speaker 2: So on that which could have been prevented. Okay, this allergic reaction which we don't know the history, it's a non-preventable but in the future once you know it, so in the future you can prevent but the dose related which can be prevented. Okay, so there we review either the dose was appropriate or no either the rate of administration was okay or no. Okay. And the dilution of the medication, we had one case unfortunately the patient who received vecan drug irritant and vesicant drug on a higher rate of administration, the patient develop extra position, skin rupture like that. So when it was reported as an adverse drug reaction came to the clinical pharmacist, he review in coordination with the nurse manager. So we find out that dilution was okay but the rate of administration was very fast. So then we find out we don't have the list of the irritant and vesicant drugs and the nurses or the pharmacists are not fully aware that is this irritant or vacan what should be. So a committee sit down, brainstorm happened and then what we did, we developed a list of the medication which are irritant and vesicant, then we develop the guideline, how it should be diluted, what should be a rate of administration and then we educated all the nurses and We distribute it.

Speaker 1: What is next?

Speaker 2: We announcement and distribute it between the physician, the nurses. So once you prepare this guideline, a multidisciplinary team they reviewed, agreed, signed and we through the quality make it control. So any procedure guideline we prepare then policy, how to manage the extra ization, what are our irritant and vecan drugs and then all these policies we make it controlled and our all policies there is online portal, we call it SAP jam. So it is there so all the staff can access this SEP jam and I mean they can review the policies also an education series of education conducted on this.

Speaker 1: Okay, nice. So from your experience to require like 12 years, how did you find the level of the knowledge, the attitude of the pharmacist toward the PV and the A DR reporting as you are working before different places? Yeah. So did you find that the knowledge level, the awareness level is there?

Speaker 2: Yes, exactly. I will tell you especially on the pharmacist level, they are very well aware if we show you our system. So first thing they have to check while medication appropriateness review is allergy or history, you cannot proceed to the next step. So there are two ways. One is the system who makes you mandatory to review this and the other is awareness and education. So we can control it in both ways, right? So when the pharmacist, they know that there is a KPI and also being monitored on a monthly basis. So they will be more aware about this that they have to check the allergy history of a patient. Also I would say because it could be a very little and toxic if any patient receive a medication to whom he's allergic. So it's mandatory that we educate our pharmacists on this about especially the cross sensitivity of different classes, reaction from one class to another. So I would say yes for sure the pharmacists are fully aware about this and they have been trained.

Speaker 1: Do You feel like the changes has to be started from the system of the hospital itself?

Speaker 2: Exactly. If it's not mandatory for them to report, maybe the pharmacist will say it's okay, I'll not do it. I would say policy and procedure is very important. If anything, if there is no policy procedure, if you are not aware that this going to be monitored, so the people will take it easy and they will not do it. Second system can help in this you can put some like the stoppers, you cannot proceed to the next level until you don't put the allergic history for this patient, right? Our pharmacist cannot proceed to the next, until he reconcile the order, he cannot bill or like that. So in reconciliation he check the previous patient medication history. Second we check the allergic history and also the system gives you a flack or it'll alert you that this patient is allergic to this medication. Also, I would suggest when the physicians they are prescribing some HIS system, it gives them allergic that this is, I mean this patient is allergic. Thirdly I would say at the level of admission or at the level of when the patient is coming to the hospital. So you are taking a history of the patient. It is very important to educate our nurses and physician also to document in the system and flag the system that this patient is allergic to some medications. Excellent. So it is like a process of chain, I mean chain process. If you break this chain somewhere it continue, it'll continue and patient will end up with wrong medication.

Speaker 1: Right. It's excellent. Very nice. So Doctor if you can say what is your suggestions to improve the ADR reporting and the PV system? Not here because Michelle, it looks it's established maybe on other sites, other hospitals how to increase the awareness between the other pharmacists because some of the pharmacists who graduated, they didn't study the pharmacos in the undergraduate curriculum. Now recently it's start to be added and I'm one of them who didn't start it in the undergraduate but I studied in master degree.

Speaker 2: Okay. So I think you see medication safety is a very important topic. Topic and the pillar in medication management and use recently you see the last week there was WHO, they celebrated the medication safety week. And I would say it's very important from the management and organization side and also for the pharmacy side because they are the custodian of the medication and they are responsible to make sure that the patient, they receive an accurate and safe medication at the end. Secondly, I would say that we can encourage the reporting only when we make sure that this reporting is non-punishment. Okay. The staff and staff, they should be encouraged enough and they should be considering this reporting is a process to improve the medication safety, improve the gap and they should not feel that they will be punished for this. Okay. Error can happen from anyone but we need to put the stoppers, it should not reach to the patient. So I would say training sessions and continuous education for all healthcare providers on this topic will enhance the ADR reporting practice. And most important, the error reporting should be encouraged and should be non.

Speaker 1: Okay. Thank you so much.

Speaker 2: You're welcome. Thank you Doctor

Speaker 1: Thank

Speaker 2: You. You're welcome. Okay.
