# Supplementary material for: A qualitative study to investigate pharmacovigilance systems in Dubai hospitals
Source: PLoS One. 2025 Sep 10;20(9):e0331929. doi: 10.1371/journal.pone.0331929 (PMC12422479; doi:10.1371/journal.pone.0331929)
Supplement: S1 File — (ZIP) [file pone.0331929.s001.zip › M2.docx]

Speaker 1: Good morning Dr. How are you today?

Speaker 2: Yes, good morning Dr. Sawsan. Thank you so much. I'm good.

Speaker 1: Thank you so much for accepting this invitation to do the qualitative interview with you.

Speaker 2: Thank you.

Speaker 1: The study will be about xxploring the PV system in ,,,,,,,hospitals. Just for you to know this will be recorded, then we'll use this record for publication later on and I already shared with you the consent form and you read it, sign it and you send it back to me.

Speaker 2: Yes.

Speaker 1: Thank you so much. So let's start. First of all, can you please introduce yourself, your qualification, you're running in hospital and you experience?

Speaker 2: Sure, sure. So my name is ……. I'm currently working as a manager clinical pharmacy at ,,,,,,,,,. The way my qualifications, I did doctor of Pharmacy back in 2009 and then I did master of science in clinical pharmacy. I also did a master in business administration, MBA in healthcare management and some other certification diplomas in healthcare management. So this is currently 13th years I'm working, this is my experience. Yeah, 13 to 14 years. So my experience has been like a clinical pharmacist towards the manager clinical pharmacy.

Speaker 1: And you got your bachelor degree from where and your master degree?

Speaker 2: My bachelor from Pakistan, my master from uk, then MBA from Dubai, but the affiliation from a university in Spain.

Speaker 1: Okay, good. So now we'll start talking about the pharmacovigilance at your working place. Is there a pharmacovigilance center or anybody assigned with the responsibility for monitoring a DR reporting in your working place?

Speaker 2: Well there is a reporting system in our hospital we have called the OVR and it's merged with the medication error portal. But not specific system for the ADR alone. ADRs and MER reporting happened whenever there is any pharmacovigilance, sort of like ADR. So they report on the same, it goes to the quality and then there is, I mean my role is as a manager clinical pharmacy, but we don't have a specific person who is taking care but I am taking care of the ADRs, etc

Speaker 1: So you don't have physically exist pharmacovigilance center in your hospital?

Speaker 2: No, not exactly.

Speaker 1: Okay. And you are the one who's responsible about ADR reporting,

Speaker 2: Correct. I am the one responsible for ADR reporting at the hospital as I am the Clinical Pharmacy manager.

Speaker 1: Okay. So is there a clear mandate structure who is responsibilities and reporting line for Pharmacovigilance Center?

Speaker 2: See we have a policy and the understanding as per my observation, the understanding about pharmacovigilance or specifically talking about the A DR adverse drug reactions, the staff awareness about this is if any allergic reaction is there, so they have to report it. So they report through the MER portal or the OVR portal and it by default goes to the quality. There is a person called the risk manager. Basically she handles all type of occurrence variance reports including medication errors.

Speaker 1: Well, we will talk about this procedure later on in more details. But is it mandatory at your working place to do the ADR reporting?

Speaker 2: About the allergic reaction? Yes, the policy talks about this that we should report allergic reaction and other serious and major ADR, but the other type of the adverse drug events, it is not mandatory. Yeah.

Speaker 1: Is there any an annual budget allocated for pharmacist activities at your hospital Specifically?

Speaker 2: No, there is no any annual budget allocated for PV activities. If there is any quality or safety awareness week in the hospital, then we do some activities related to PV in that week. But not putting any budget specifically for pharmacovigilance, no.

Speaker 1: Can you please share with us your experience with ADR reporting either in this hospital or your previous experience in other hospitals?

Speaker 2: Yeah, so talking about the ADRs or adverse drug reactions as I was explaining before or adverse drug events, the understanding and awareness here with the staff is to report if there is any allergic reaction or flu reactions. And my experience seeing this is very under-reporting, the numbers are coming one, two or three per month, which is not the real case actually there are many adverse drug events related or adverse drug reaction which goes unnoticed and not reported.

Speaker 1: So the patient or the physicians themselves do the reporting?

Speaker 2: Yeah, The reporting comes mainly from the nurses. Okay. Because they are the persons administering the medication so they observe but it can come from the patients themselves. But here at this level the patients are not involved. Okay. I didn't see any reporting from the patients. However they should be involved, I believe in this and the physicians very less likely to report it mainly are the nurses or if the clinical pharmacists are involved and they review the patient cases. So they are also one who can report it.

Speaker 1: Okay. So not any specific cases happened recently with you in the hospital other than allergy reaction happen?

Speaker 2: The reported one, yes. Still now there is no other ADR been reported considering other factors? Factors except the allergic reactions.

Speaker 1: What about your previous experiences in other hospitals

Speaker 2: Also the almost same.

Speaker 1: The same,

Speaker 2: Yeah. Medication error. Sometimes there are, I mean also medication error focus on wrong prescribing dispensing or wrong administration. But I feel a gap on monitoring effects which also an important part I believe of pharmacovigilance. So that's missing And what's been reported only is the most of the time anaphylaxis allergic reactions or extra positions. That's it

Speaker 1: Like simple things on?

Speaker 2: Yes.

Speaker 1: Okay. So now could you provide us please with the details explanation of your hospitals ADR reporting procedure? The one you started talking about it before, how it go es exactly?

Speaker 2: Okay. So when any ADR happens, we have the medication management tools policy. There is a policy on medication administration. So nurses are oriented and competent about the medication administration on seven rights of the patient, what they need to check before administering and the same for the pharmacist before dispensing. And also seven elements of order verification for example for physician before prescribing that is the right medication for right patients, et cetera. So when the nurse is the person who is administering the medication for example, so if she finds any complaint from the patient's side, for example, patient complains after administering some sort of itching or irritation. So the first thing she does stop the medication, report to the doctors and stabilize the patient if it is required. And once the patient is stable, so then they have to report it through the OVR portal. Once she completes the OVR portal, it goes to quality and the risk management department, which is called QRM, there is one person risk manager assigned for all sort of errors.

Speaker 1: Is she pharmacist or physician?

Speaker 2: No, she is I believe is a nurse but she did the qualification further in the quality related. So she is not a practitioners as a nursing licensed or the pharmacist or physician but she is a quality person then if it is related to medication, for example in a DR, so she assigned to a person in the pharmacy and also to the HODs who's assigned for the occurrence area for example. So I am the person assigned in pharmacy, she send it to me and also by default it goes to the pharmacy director also but to review and then get back to them.

Speaker 1: And who's taking the decision of the action has to be taken from your side or from the quality?

Speaker 2: So we investigate and we let's say, so it's called investigations and the putting an action plan to prevent other suggestions and then it goes to the quality and they're the one then putting the actions in plans.

Speaker 1: Okay, so you are the one who put the action on the plan then it'll goes to them and they will decide at their level the quality? Exactly, yeah. What about if the patient do the reporting himself or herself?

Speaker 2: No, I didn't

Speaker 1: See pharmacist patient.

Speaker 2: Yeah, they have an access to the patient experience feedback.

Speaker 1: So

Speaker 2: Might be they can do from there or if it is an ambulatory patient for example. So they can also provide the feedback but they do not have an access to the OVR portal so they can give their feedback through the customer feedback for example, or directly speaking to the physician or the nurse or for example if there is any follow-up done with the patient. So they can explain sometime if the pharmacist for example giving a follow-up so they can update what type of the side effects for example, if we initiate such questions so they can update.

Speaker 1: So all pharmacists and outpatient they have access to this system to do ADR reporting? All of them,

Speaker 2: Yes. All of them.

Speaker 1: Okay. What about the workflow of ADRs reporting to national level? Is there continuous reporting of these ADRs happened to Ministry of health or nothing between hospitals and minsitry?

Speaker 2: It's not very clear to be honest. I have seen one memo in the beginning initially. So we are under the Bay Health Authority and there is one circular mentioning that we need to report the ADRs and there is the timeline. So when you click on that link, so it takes you to the ministry of health link, which is a national level, it was like two, three months back. But now I've just noticed again the link is not working. So when I ask from the quality, so they are only reporting the medication errors, which is the required KPI from the DHAE. Okay. So I don't think so there is a DR being reported across the hospitals.

Speaker 1: Even the serious A DR reporting happen Not reported,

Speaker 2: Yes, but there is a portal in MOH where we can report, but I mean it's not mandatory what I meant.

Speaker 1: But at Your working place, it's not mandatory to keep sending a continuous report to of ADR?

Speaker 2: Yes, they do report medication errors but I don't see ADR.

Speaker 1: So they do the reporting of medication error to them?

Speaker 2: Yes. Medication error being reported to DHA, this is,

Speaker 1: is it like a continuous process that has been done every month, every two months or every week or every time it's happened on the same time they have to send it to them or what?

Speaker 2: No it's not like that. So they do I guess on monthly level or the quarterly level. So I mean we report if any medication error happened in the hospital internally within 24 hours to 48 hours and on a monthly basis we have a key performance indicator, let's say how many medication error happened and we report it to the quality but the quality then further report to DHA on I think a monthly or quarterly this I need to verify.

Speaker 1: Okay. So Dr, from your review what might pharmacists face when it comes to practice PV and reporting ADR? As my study is concentrating to hospital pharmacists.

Speaker 2: So the obstacles I would say in most of the cases is the poor communication amongst medical professionals. For example, if I talk about outpatient or inpatient, you are not dealing directly to the patient at the level of administration of medications. So if we have a clinical pharmacist they are having around so they can know directly if any sort of ADRs are happening, if it is missed from the nursing side. So the pharmacists cannot know. So there are some obstacles you have to go through the patient profiles exactly find out there are some trigger tools, maybe trigger medication from where you can find out. But otherwise you will not be knowing if any adverse reaction happens to the patient or unless you have an access to the lab reports from where you can find out.

Speaker 2: But so this requires really big time to review the patient cases which most of the pharmacists in their busy routine will not find this time. So the time management here to review the patient cases to find out if there is any adverse drug reaction is a big obstacle. Second, the reporting of the medication errors or ADR on the OVR portal, sometimes we as a pharmacist feel that it's taken as a personal between the team as well and being worried from the consequences. So why did we make O-V-R-M-E-R? So the encouraging here to the staff or to the team that it's to improve the process of medication safety, not on the personal level or to punish the staff. So this is also I feel an other obstacle between the team members to keep the positive relation not to report the things. So this is I believe second thing,

Speaker 1: Yeah, please go ahead. Complete

Speaker 2: And the busy timing which makes also the reporting very less so why I have to go to the OVR portal, fill all the details so then explain all the things. So this is another obstacle to find a smart solution, less time consuming to report it could be another solution to increase. So this is another obstacle.

Speaker 1: Yeah. And do you think all of them also are aware of process of reporting ADR or this can also be barrier for them? So what is my observation?

Speaker 2: Yes. We have a team of the pharmacists and I believe in other hospital would also be like this. So some of them are not aware that they have by default access to the OVR portal whenever here the management they did, they given an access by default but you have to activate it. So might be there is an orientation issues or some pharmacies they are not aware that they have an access to the OVR portal. Another confusion. They feel like either they have to report it or they need to report to their manager or the senior person they can report it. So also again sometime finding not a manager around you, you will miss a chance to report these things. Alright. So I personally observed some when I asked the staff to report it and they said we don't have an access to the OVR portal.

Speaker 1: And do you think also is there any barriers at the managerial level or from the administrative level that can prevent ADR reporting?

Speaker 2: Can you repeat again the question? Sorry.

Speaker 1: Do you think that also there are any factors that can affect ADR reporting at the managerial level or the administrative level that to prevent pharmacists from doing the ADR reporting?

Speaker 2: Yeah see could be, there are some factors could be as I said, when you are investigating any OVR or the A DR reported, so let's not bring in the factor of blaming or let's say or the factor of making some person responsible that this error happened or this A DR happens due to some factor. Let's say, I will give you an example and a DR happened due to the wrong rate of medication administrations, okay some ADRs, okay you cannot prevent but some ADRs you can prevent it. It could be affected like the fast rate of drug administration. So during investigation you found that this A DR has happened due to a fast rate of administration. So directly your mind will go towards who administered this medication, who set the rate of administration. So the managers or the administrator level should not think or should not bring these factors.

Speaker 2: I think these are the factors which should be taken out. So what they should think, okay, the wrong rate of administration is the root cause of this A DR. So what are the factors that this has been given on the wrong rate? For example, you can find is there develop the guidelines about the administration of medications about some sort of like these medication which can cause extra ization or infusion related reactions. So find out if there is no guidelines so you need to develop the guidelines or if let's say there is a guideline but was this staff been oriented on this or there is no orientation records. So I believe this could be some of the barriers if you investigate in a way that the staff becomes responsible for that, not the process because you need to improve the process not to look into making some person responsible. So this feeling of fear will make the staff not to report.

Speaker 1: So here's my questions now from your standpoint, how we can encourage the hospital pharmacists to do more ADR reporting and to practice PV?

Speaker 2: Yeah, so same answer goes like to let them believe that this reporting or the A ADRs reporting is to improve the process, not to make someone responsible and blaming also they need to be realized if they did not report any problem, this will go unnoticed and the errors will keep on happening and there will be no actions, plans will be in place. So this is one to realize the pharmacist, the importance of reporting and if it's not reported it'll go unnoticed and there will be no improvement. But once any A DR or error has been reported, then there should be a proper investigation or action plan, not only some comments and then at the end nothing has been done. We did not find out the root cause for example and there is no prevention measures has been taken to not happen it again. For example, nowadays there are many AI tools for example or for example if you find any error or adverse reaction happen due to drug drug interactions.

Speaker 2: For example, if any one antibiotics given with another let's say a OME for example given with the quinolones or azithromycin, it'll cause QT prolongation and this could lead to adverse drug reaction in the system. The system should support the alert on this drug drug interactions to the physicians or the pharmacists when they're prescribing or dispensing, which could be one of the solution. So coming back again to the question. So giving them self-realization that their reporting can improve the process of medication safety, the patient outcomes and if they have reported this will be taken seriously and the process will be improved, not the persons to be made responsible for these things.

Speaker 1: And do you think if there is a specific educational module or continuously training given for pharmacists can improve the A DR reporting?

Speaker 2: Yes, I believe Training sessions, continuous education is very important here. Even if you have given one time then you need to keep them realizing the importance of reporting by continuous ongoing education. This will definitely improve the reporting. I have noticed myself, so one time the staff were oriented, in that month some good reporting happened and then later on when there are no follow up educations, you will see that the trend of reporting going down. So you have to keep on educating alerts and also when the new staffs are coming, the new manpower you have, the new human resources, so they need to be oriented also to make themselves aware what is the policy on the pharmacovigilance and the reporting etc especially for the people came from outside the country.

Speaker 1: Yeah. And how might, do you think that a multi-stakeholder approach involving all healthcare providers collaboration together also can improve the A DR reporting including the physician pharmacists nurses?

Speaker 2: Yeah, it is very important. As I said for pharmacovigilance is a multidimensional and multidisciplinary approach. For me vigilance happens at the level of, let's say you can say the root cause goes towards the prescribing side also. So who is prescribing is a physician at the level of dispensing. So who's dispensing is a pharmacist at the level of administration. So who is responsible is a nurse. So involving this triangle; Physicians, nurses and pharmacists is very important to improve the process of pharmacovigilance. And the reporting can happen at the level of prescribing itself, dispensing and administration and even the patient themselves

Speaker 1: And The patients?

Speaker 2: Yes, exactly. So when the patients and the family for example also be involved in ADR reporting, this can enhance the PV activities. So they are the one taking a medication for example in ambulatory setting. So if they find any unintended side effects for sure the patients are consulted at the level of dispensing by the pharmacist, answer their curies expected the side effects being informed. But if there is an unintended side effects appearing to the patient so they should get back to the pharmacist or to the physician and they should report it.

Speaker 1: Okay. So from your review Dr, what kind of studies are we going to need in the future to improve the reporting of ADRs by UAE healthcare providers in general?

Speaker 2: So I believe the trend and types of most ADRs happened should be reviewed. For example, it should be monitor what type of the reporting is being done and then what we are missing in the reporting having attended some many audits of the healthcare quality. For example JCIA, we have A SHP. So there is always a comments about underreporting of ADRs and there is always a suggestion how to improve the adr. So the awareness about the different type of the adr, what is the difference between medication errors and ADRs? What is exactly the form? EIG is very important and see what type of the trend of the reporting is there. So I'm not sure about other hospital could be different, but my personal experience either it's happening the medication errors you are reporting, which can happen at different levels or you are reporting allergic reactions and we are missing the unintended side effects part.

Speaker 2: For example, a patient has been given a dose of insulins and he developed hypoglycemia. So this is unintended side effects there requires an appropriate dosage to be administered. So this should be reported some patients being given anticoagulants and you find blood in their stool or the patient start bleeding. This is also I would consider as adverse drug reaction but I never seen it's been reported. So a patient's admitted in the hospital on excessive antibiotics and he developed C deficit for example infections. So this should be reported as a DR because antibiotics induced C deficit but nobody took the reporting. So nobody reports it Here the education is missing. Awareness should be done. And again I said we should find smart ways of reporting. So we need studies that highlighted this point and give suggestions of using technology to improve reporting

Speaker 1: Using technology you mean?

Speaker 2: Yeah, use of technology, a dedicated persons or center where you can approach to a person for example, most of the time the patient, the staff don't report because they are busy, they don't find the time. So we need to find the different ways of finding a ways to smartly report it. Most of the hospital either they have a paper form or online form, which is a very lengthy form and the staff do not find the time. So finding to the point forms or what you can introduce. And we are thinking also about this to introduce a medication safety hotline or like a hotline for the vigilance or dedicated person. So for that you need to assign a dedicated person, you can name him anything medication safety officer or forec vigilance officer for example. So simply you have a hotline when you find any A DR. So simply you pick up the phone and introduce yourself, your area and precisely explain what a DR you focus and then leave it to this dedicated person who will hear this message, will transcribe this A DR and report it on your behalf. So I believe this will increase the reporting and here the support from the administration management is required and eventually it'll give you a safe environment for the medications usage and positive patient outcomes. I think this is the area where we need to study more and been neglected.

Speaker 1: Yeah. Okay. Thank you so much Dr, for answering all my questions. To clarify it for me more about the BV system at your place. Anything else you want to add at this point about the BV and A DR reporting?

Speaker 2: Yes, I would say also from the government level, from different states to different states, the responsiveness in UA is different. This is my personal opinion and based on my observation, without naming any authority, where you find that you've been mandated to report it, not only the medication errors, also the adverse drug events, they name it now adverse drug events or adverse drug reaction, which is a bigger umbrella and it contains everything. And they ask from your side the action plan what you did to prevent them and they ask for a dedicated person to whom the contact person and usually it should be a medication expert and most of the time it is a pharmacist. So they ask them to review your ADRs, put an action plan, give it back to the authority. If you don't put this action plan, they follow up and also they have an active team to follow up on your action plan so that the trend of the ADRs can be sorted out in a positive way but in somewhere maybe you will not be mandated. There is no active follow up. So which will make the staff feel not to report Nothing.

Speaker 1: To encourage them to report.

Speaker 2: Yeah. So there is nothing.

Speaker 1: Okay. Do you think that if we put an incentive for the pharmacy can encourage reporting?

Speaker 2: That is my next point I was about to talk. It happens in some of my previous work locations, putting in incentives will encourage the pharmacist to report it. We have in our hospital of pharmacist intervention system where they put the most of the type not only about the ADRs but any type of the interventions they did while reviewing the medication orders. So we, we recently started putting a best intervention reporter or a documentary and we started to select on a monthly basis. And this is at the department level which, and to give them some incentives on this.

Speaker 1: Do you apply this at your working place now?

Speaker 2: Yes, at the department level, yes. But however, if it goes across the hospital level and from the quality side they can name it quality champion or quality on a quarterly basis. So I believe it'll increase the reporting and specifically dedicated to pharmacovigilance considering the medication safety and the patient outcomes.

Speaker 1: Very nice, very nice. Thank you so much Dr. Kar, for your timing and for all your valuable points and to your review about the A DR reporting and hopefully we can do something to improve this ADR reporting in the future inshallah.

Speaker 2: Sure. Sure Dr. So thank you so much and I believe yeah, the different stakeholder needs to come forward, collaborate, and look forward for the medication safety. Thank you so much for.
