# Supplementary material for: A qualitative study to investigate pharmacovigilance systems in Dubai hospitals
Source: PLoS One. 2025 Sep 10;20(9):e0331929. doi: 10.1371/journal.pone.0331929 (PMC12422479; doi:10.1371/journal.pone.0331929)
Supplement: S1 File — (ZIP) [file pone.0331929.s001.zip › M3.docx]

Speaker 1: Welcome Dr. How are you? How is everything?

Speaker 2: Everything is well.

Speaker 1: Okay. So first can you please introduce yourself?

Speaker 2: I'm Dr. ,,,,,,,. I'm a medical director of hospital pharmacy.

Speaker 1: Okay. How many years you are working as a pharmacist?

Speaker 2: As a pharmacist? More than 30 years.

Speaker 1: 30 years. And which country did you graduate from?

Speaker 2: From ,,,,,,,.

Speaker 1: Okay. And do you have a master degree or only bachelor's?

Speaker 2: Yes. Master

Speaker 1: Master. In what

Speaker 2: Master in Pharmacology

Speaker 1: So what is your experience with ADR? Reporting? Reporting? Is there any ADR reporting at your hospital?

Speaker 2: Yes, of course. We have in our system, in the port we have, if it's happening here, we have to submit it. The communication of our hospital pharmacy.

Speaker 1: Okay. So can you just explain for me the flow of reporting process? The rate of this is the ADR reporting. How is the system for reporting?

Speaker 2: Yeah, we have in our system that we call the portal, portal is open for all the staff. There is a different type of portals here, but we have a specific one for address drug reaction. So that whatever is happening in the ward, in the pharmacy, whenever they have the person who's responsible for that, he must apply for this portal, this transaction.

Speaker 1: Okay. And then this reports came to the pharmacy.

Speaker 2: Oh, all the system. Everyone in the hospital, they can see it.

Speaker 1: Okay. So who's responsible for this one?

Speaker 2: To check the ADR activities, of course the head of the pharmacy is the person in charge. But after that the medical director and then in the ward, if the ward there is a metro responsible for adapting after that will be discussed immediately.

Speaker 1: Okay. So in the wards they have their own system they were Reporting?

Speaker 2: No, no. System is the one system.

Speaker 1: Okay.

Speaker 2: System is the one system. If there's a patient, it belongs to the pharmacy. Of course they report it'll immediately come in the alarm direct here.

Speaker 1: Okay. And then who do that?

Speaker 2: Then we take the action. Me responsible for that.

Speaker 1: You would decide that. So you are the one who responsible for ADR. Okay. Excellent. And after that, what is the action?

Speaker 2: I am the one responsible of ADR reporting system at the pharmacy as other pharmacists don’t deal with this system directly and they don’t know how to do reporting. The problem is can be solved with the pharmaceutical. So otherwise we have the medical and there is a committee we discuss and action immediately.

Speaker 1: Okay. And do you report it to DHA? Is there a connection?.

Speaker 2: If it's need to record, like serious one, then yes, of course we reported to MOH.

Speaker 1: So when it's needed to be reported,

Speaker 2: Of course if is the patient complain, if the patient passed. If the patient passed, of course they will contact. So we'll explain to them which action we took.

Speaker 1: Okay. So there is no regular reporting for the DHA whatever happening?

Speaker 2: No, we're not reporting. The medical director is reporting if they need anything. They're reporting. You are not reporting from our pharmacy. No report for DHA.

Speaker 1: Okay. So there is no connection with the DHA?

Speaker 2: No, there is a connection. But regarding these things, we don't have any relation. This belongs to the hospital of course. So that this is through the medical director that we with dh.

Speaker 1: So I can ask how many ADR reports per year you are reporting visually? Like plus year?

Speaker 2: The good according to the Canadian accreditation, which we are following here, we are reporting every single thing happening. We have, it's too much. Whatever is happening, we're reporting every mistakes happening. Even small mistakes. But we have to report it.

Speaker 1: It's like if we need to put a number, can we put a number?

Speaker 2: I cannot say, but we have too much. Too much. Too much.

Speaker 1: So

Speaker 2: It is maybe monthly one time that if I'm telling that every month we have two or three like that, the average like that. Okay.

Speaker 1: Okay. So did you take any courses for ADR reporting? Do you teach stuff?

Speaker 2: No, it's because I'm almost more than 20 years in this field. From the experience we are doing everything like this, no need to take it first.

Speaker 1: Like the other staff, the nurses in the ward, are they taking any training courses for any other?

Speaker 2: Yes, of course. We have two times per week. We have what's called morning report, discussing everything and updating everything's happening, inform them.

Speaker 1: So do you have any comment, any advices to improve the ADR reporting pharmacovigilance system in the hospitals?

Speaker 2: In general, I'm very satisfied from our system and expectation, especially in the Iranian hospital, we are very strong that the committee is very strong and so we are aware about everything because everything must be reported. Otherwise, if anything happened, the person is responsible to report will be punished so that all they know About.

Speaker 1: So have there is a policy for not Reporting?

Speaker 2: Yes, of course.

Speaker 1: And everyone is responsible for reporting.

Speaker 2: everyone has to be responsible for reporting. Nurses, pharmacists, physicians,

Speaker 1: And I mean what is the center now of reporting for Different departments in the hospital?

Speaker 2: Of course all different department are already reported to the medical director.

Speaker 1: To the medical director?

Speaker 2: I'm a pharmacy medical director.

Speaker 1: ooh, you mean pharmacy, medical director. Okay

Speaker 2: So everyone reporting to pharmacy medical director.

Speaker 2: And he's taking the decision. And he decided the action to be taken or for example, if you are here in the pharmacy for this, if belongs to the pharmacy, action must be immediate action.

Speaker 1: So you are the one here who decided?

Speaker 2: Yeah, if it's passport pharmacy, of course the medical director will take the action required action

Speaker 1: From your view doctor, how we can enhance ADR reporting at the hospitals level?

Speaker 2: First providing continuous education about ADR reporting for all healthcare providers. Then, give a rewards or incentives to the one who is reporting the most on monthly basis. Assign budget for the PV activities. And finally, encouraging the patients themselves to report can enhance the ADR reporting practices.

Speaker 1: The last thing I would like to ask you, what kind of studies do you feel that we need in the future to improve reporting of ADR in the UAE

Speaker 2: Maybe to find the obstacles for underreporting in the country. What are the difference between ADRs and medication errors.

Speaker 1: Okay. Thank you so much, doctor, for your time and for your information. Thank you.
