# Supplementary material for: A qualitative study to investigate pharmacovigilance systems in Dubai hospitals
Source: PLoS One. 2025 Sep 10;20(9):e0331929. doi: 10.1371/journal.pone.0331929 (PMC12422479; doi:10.1371/journal.pone.0331929)
Supplement: S1 File — (ZIP) [file pone.0331929.s001.zip › M4.docx]

Speaker 1 ([00:00](https://www.rev.com/transcript-editor/shared/kBAEAStvbarhuxsA05RcY_J8q25CP0sub2QDlY06T8GV5iE4dyfQmk3f8I7Df7qIDIUH7C2TfzGeY38Oz4g25OijzkA?loadFrom=DocumentDeeplink&ts=0.69)):

Okay. Good morning Dr. How are you today?

Speaker 2 ([00:02](https://www.rev.com/transcript-editor/shared/rO5gc1TYeV_yuOLb34kk2YoBgZWM2EBCZ4X-yIHvcLkG9JLHkVa8ID8vuzZsc8hQ-L7eHQO1vKxg1x2D32sueP23cAg?loadFrom=DocumentDeeplink&ts=2.98)):

Good morning. I'm, fine

Speaker 1 ([00:04](https://www.rev.com/transcript-editor/shared/96IFX7Zt1XtlyCpEzCDJRLufMiSWyvhz0X7TMhWQXWqh8gllLFWlgwVFPjpq6OK1H9IT6IvUDX8O59OFEMG1IW0MuQk?loadFrom=DocumentDeeplink&ts=4.59)):

If you don't mind, I will conduct with you an interview for the qualitative study of PV system in different hospitals in Dubai. And this study aimed to evaluate the PV system and ADR reporting among the Dubai hospitals. So first of all, can you please introduce yourself?

Speaker 2 ([00:25](https://www.rev.com/transcript-editor/shared/NTpx3JcpSvXE9oeQJfmzdoqqSR9XFQMOpK658XE9VYyA8Uwlv4bFhQPs_SU2pu-WB44Fx7_bSCR8bs0AsgV6QLfzhiM?loadFrom=DocumentDeeplink&ts=25.02)):

My name is ,,,,,,,. I have a PharmD degree. Graduated in 2008, start working as a clinical pharmacist since that time in 2008. Started with neonatal ICU and parental nutrition. 2013 I become a clinical pharmacy specialist. I was responsible for pediatric service or clinical pharmacy for pediatric population. Then I moved to Dubai in 2018 to be the assistant director for clinical pharmacy service, which under title of clinical pharmacy specialist. In 2020 or end of 2019, I become the acting head of pharmacy department who was responsible for the clinical pharmacy service and operation services.

Speaker 1 ([01:17](https://www.rev.com/transcript-editor/shared/sVG52BngyfT1dFjORSQuxCFf7Py67pSkBMRq1GJBZEKVE3nkJxU5ZP-ukAT-gMRvDZURkdDxWg2GXFLoWEbCYoz6vO4?loadFrom=DocumentDeeplink&ts=77.28)):

Okay, excellent. So as you said, you have a PharmD degree and you continue in the clinical.

Speaker 2 ([01:25](https://www.rev.com/transcript-editor/shared/uGhgUd8z7dkwU2iFtDB3HCcXjtpuh14yAJ7uX4zWtFrqwJdGuyPBn5spe2FHUHGwntCA_O95MmXxk6vmH-6ayQUn_oA?loadFrom=DocumentDeeplink&ts=85.25)):

Yes, and I have also a master degree in pharmacoeconomic in addition to other certificates like medication safety, board of American Board of Nutrition, pharmacist, addition to other qualifications.

Speaker 1 ([01:38](https://www.rev.com/transcript-editor/shared/Fc1mcEqU2IJz8uq61eS3U58BymioQf9WMOXYg3fbw817O4wWfYcoUdkyhIeHL4vn2YEdaLDPi0ve2nqCKsSJZ1SaZpc?loadFrom=DocumentDeeplink&ts=98.97)):

And this PharmD, you got it from Jordan?

Speaker 2 ([01:41](https://www.rev.com/transcript-editor/shared/F-gRvaWTm08K7PfqiELfZfRNjigaHCNW2wYvDs8UH5J6E3EYY3wDZVCO1wUAdccMtIjYvEo_JWIXkQEhVXv85F5KSHk?loadFrom=DocumentDeeplink&ts=101.58)):

Yes, from Jordan University of Sales and Technology in 2008.

Speaker 1 ([01:46](https://www.rev.com/transcript-editor/shared/Euxyzes80tTZnMyNgJ2OuTMtTCCpaLazHdbP6_JnqitK7Xmt3WrOK9nGq_kp50ZEHHf7RNPtimqCjjvVgk1eWggvQ7I?loadFrom=DocumentDeeplink&ts=106.52)):

Okay. Okay. Can you please tell me your experience with ADR reporting as you have now long years of experience here in UA?

Speaker 2 ([01:56](https://www.rev.com/transcript-editor/shared/CKed7QVTMWJ8KNbrSP-BsD4bGCipxkLRvqawnWmOetw0AtDKDz7-BDkspM9K-wkBI4G3BVVNl9Rnqr6vrtf7wBKGWPo?loadFrom=DocumentDeeplink&ts=116.19)):

Okay, first of all, the main challenge is understanding of meaning of adverse drug reaction for a different clinician to encourage them to report it. Yes. We started in first workplace with the nurses, with other healthcare providers, the meaning of ADR and the aim of reporting.

Speaker 1 ([02:20](https://www.rev.com/transcript-editor/shared/8ChPU-lo9-Axv20sNSgMlPWO6m6c20ZuEyaUEppfsnSn7eY6pbGEGyw8jRm5r_O81GSf-OBho0UnACsFNZInHi8WHyw?loadFrom=DocumentDeeplink&ts=140.6)):

Okay.

Speaker 2 ([02:25](https://www.rev.com/transcript-editor/shared/vjbIRUskTpchLB1Rl--1UG6aoo-fdvT1YfSRy7GHwHL47Kv8DMKpiRs5yqOfTT9-5O9HGYv9mcGJgC5d9p3wyS1sujo?loadFrom=DocumentDeeplink&ts=145.04)):

So to introduce the meaning and the aim for the nurses and Physician, whoever the pharmacist. Pharmacist as well. If he receive the report pharmacist, and it's not always providing direct patient care like a clinical pharmacist. Usually they get reports from the patient asking is it related to the drug or related to the disease. And then the pharmacist can evaluate from that time is they make this specialty of reporting system to the pharmacist. So what we do since 2008 until today, we encourage everybody to report regardless what is the events and then the pharmacist will do the further investigation to classify it as medication error, adverse drug reaction, et cetera.

Speaker 1 ([03:08](https://www.rev.com/transcript-editor/shared/m-G0w8M-qt4Ec94v_wknc8htK2djnSFrzQx3NRjdyitSTInXKg30b0yucAoz-Jb_5s_MdFCdj211dXrcveN14ybCA7A?loadFrom=DocumentDeeplink&ts=188.26)):

Okay. So you are encouraging your healthcare provider, nurses, physician, other pharmacists themselves for the report?

Speaker 2 ([03:15](https://www.rev.com/transcript-editor/shared/knzmJWeFg7jxhPJ_QAGDz1Loukk6IeoqzdmwoE9Rbnmi4M235NNhSN-jG-saIiQ0NuASFhSfmiulx4R-DZsLiCLWMNA?loadFrom=DocumentDeeplink&ts=195.04)):

Yes, we encourage all healthcare providers at the hospital to report anything related to medications. Any type of ADRs and medication errors regardless if they are major or minor, rare or common. Then further investigation will come through the pharmacy.

Speaker 1 ([03:24](https://www.rev.com/transcript-editor/shared/FF8UB755PEhKl4H8heyHiAYpKrlgmIdoMUJz7jEy3hspB6ozo47lJ58T3omfIalnItUhWU91j9JKf2YBgA_EHjYL2rc?loadFrom=DocumentDeeplink&ts=204.11)):

So okay, here the question, do you have a specialized pharmacist in your department for the ADR reporting and the PV system?

Speaker 2 ([03:31](https://www.rev.com/transcript-editor/shared/5iqVAvaDRr3u91Ri65Icj18Pj1yRSqHCY9V_5h2wyTk7HbDPZTvQHiV1lxjBGPHeT7z3AbXUMW8BS_9k6z2ufOpRinc?loadFrom=DocumentDeeplink&ts=211.67)):

Yes. Previously in my previous hospital, we have medication safety officer who is responsible for awareness and evaluation of any events. Now in this hospital, I'm doing this service in addition to other clinical pharmacists. What we do, we are the contact person for anything related to the events to guide the one who is the observer, how to report and for us what to investigate to evaluate or to classify the issue that is related to the drug itself. Modified, not modified, is it error or It is only drug reaction. So it is now under a clinical pharmacy service. Nobody like dedicated only for this service, but it is done by the clinical Pharmacist.

Speaker 1 ([04:19](https://www.rev.com/transcript-editor/shared/Otz5g3yrnbeiZzFPIoaCKNbmDzAJCEkr3y2XaKqa0T99QCVEXAosEbicZyuUBVJFL7-oGHiJmjRVv62smEfOO71nKP0?loadFrom=DocumentDeeplink&ts=259.4)):

Okay. So for example, for the outpatient pharmacist, if the patient came and he has a complaint, ADR or medication error, anything related to his medication happened, so he'll inform the pharmacist? what the pharmacist will do, is there a form he has to fill it online paper form or he came to you first and asked you what is the process

Speaker 2 ([04:43](https://www.rev.com/transcript-editor/shared/C6VKxu__XazJilukB5F0y5DKduvO6xwXmHYPrF5BBMvY1JYUVPPS7IB7MVPJRVp1KeM-h6shi1f6hWBrxi5C24i1ePo?loadFrom=DocumentDeeplink&ts=283.24)):

Here in Jalila we have RL system, which is events reporting system. It's related to any events in the hospital. They have medication part, like if it is related to medication, start reporting that this is medication related issue and the one who report will not classify what is it? He will just mention the severity of the events would happen. Like it is cause a reaction to the patient, no harm for patient, different classes from A to H, this is A to H system and it'll reach to the head of pharmacy.

Speaker 1 ([05:17](https://www.rev.com/transcript-editor/shared/sZvfNQvW9BEUAisw4WEdetKsdYD-qASEBaCSNEyp7Z9drrjFNU37iE7DGO3z4Q3nV9Ftw9Z38wELrRcuoqz3W78k8bU?loadFrom=DocumentDeeplink&ts=317.43)):

So they report to you directly?

Speaker 2 ([05:18](https://www.rev.com/transcript-editor/shared/AUAgNQoscpufytMJKxOUbpoVDa8ELuIjnMcSMWkrA5U7gyHdpTXB9lMQTWBRTtrSk0PYIuak4GN9BvwqDrLWaL015yU?loadFrom=DocumentDeeplink&ts=318.01)):

Exactly. So, my role will be further investigation is needed, further monitoring is needed, et cetera in addition to prevention of reoccurrence plan or the management of this event.

Speaker 1 ([05:33](https://www.rev.com/transcript-editor/shared/ytQPLyFSAUyPyvwMuMbhNk8fsSW2EZjDLKg_VShSfDXD3PQOSgF4RswWIyF7QzVOWrQzAtFZOHauOpBU7MJaxFIgj70?loadFrom=DocumentDeeplink&ts=333.96)):

Okay. And then you are the one who make the analysis and the decision for what should be taken now?

Speaker 2 ([05:18](https://www.rev.com/transcript-editor/shared/AUAgNQoscpufytMJKxOUbpoVDa8ELuIjnMcSMWkrA5U7gyHdpTXB9lMQTWBRTtrSk0PYIuak4GN9BvwqDrLWaL015yU?loadFrom=DocumentDeeplink&ts=318.01)):

Yes, I am the one

Speaker 1 ([05:33](https://www.rev.com/transcript-editor/shared/ytQPLyFSAUyPyvwMuMbhNk8fsSW2EZjDLKg_VShSfDXD3PQOSgF4RswWIyF7QzVOWrQzAtFZOHauOpBU7MJaxFIgj70?loadFrom=DocumentDeeplink&ts=333.96)):

Okay. So you'll do the analysis and the decision.

Speaker 2 ([05:45](https://www.rev.com/transcript-editor/shared/-a7wjmWMzJbKUeSCJ34WtLz6ZEEr47rmrPG5ZPFV1aRI_6DdDJx4Fst6ocjXoiiH8d2ahRTt0JKC4M1lzFZtjr1Rlts?loadFrom=DocumentDeeplink&ts=345.37)):

Exactly. this one will be reported to quality and patient safety department After I decide

Speaker 1 ([05:50](https://www.rev.com/transcript-editor/shared/9Oy0hFne8cABgeKMvmW8jsez00RPbv4KI_MWnaN6EiBzNnU6WvB4qIuTstFoo2vysI3NprePaH-vFLpWKomBkyxaAew?loadFrom=DocumentDeeplink&ts=350.83)):

Okay, after that you reported to ministry?.

Speaker 2 ([05:54](https://www.rev.com/transcript-editor/shared/jcbTORC7dOrCIvPIZlAyuzPOzn-LSRcuGWF233tBixyYaKVfPExSBncu5hyYKqkgObNKlL8Pb89rgHtUJjgNGDcrz70?loadFrom=DocumentDeeplink&ts=354.22)):

So quality and patient safety, they have also another analysis they need to do if they need to do further analysis like root cause analysis or any other plan from their side, either investigation or action plan, which is the highest level of action, like they reported directly to medical director or pharmacy and therapeutic committee, if there is anything to be escalated or if it will be at departmental level, they will follow up about the plan that is created by pharmacy department.

Speaker 1 ([06:38](https://www.rev.com/transcript-editor/shared/JtqfOsnvTy0A4WC0pZmm6irafj0Kggyxb6jDS0X-w76Mk7QlOUa3_llRY-f3n0GT2Q58l6Juc-5qo_rZtAfuqm68Q4w?loadFrom=DocumentDeeplink&ts=398.76)):

Okay.

Speaker 2 ([06:41](https://www.rev.com/transcript-editor/shared/QOAWHc1WtKt8T6KP6ZvVsOepaip7sY_eCESvfokgFO9_Ca6U7t6PPYzUHTOhrWlLn62XnlHdWPgaj85KjJmtp5wEyaQ?loadFrom=DocumentDeeplink&ts=401.21)):

Pause.

Speaker 1 ([06:42](https://www.rev.com/transcript-editor/shared/UdLrPWttkeceL-7YEUye8c8QJ8RlWJwFE1msc_C5ghfgsi-s6PpeETudG5TIcbJ35kYSABOwU5sYc34F2_-fTtsj-U4?loadFrom=DocumentDeeplink&ts=402.64)):

Okay. So now after reporting to the higher management, are they distributed between the different departments, the hospital, the pharmacies that happened with this patient? So be careful for the next time,

Speaker 2 ([06:55](https://www.rev.com/transcript-editor/shared/0lHKarPz-euFqIX4bK8Y3P4NbA0i7XXdGzVsAMXrq1SkSIe_lET83-DmMoGo_wjeNJtcmM98r2s_lA0-01lEQ8y99Nw?loadFrom=DocumentDeeplink&ts=415.94)):

Yes. If there's something that needs to be considered, we have a communication at hospital level. If there is any action plan done or if there's something needs multidisciplinary action for nurses, for physician to increase the awareness to make sure that everybody aware about the new plan. Yes, we do that if needed something like department, not intra department including for all departments

Speaker 1 ([07:24](https://www.rev.com/transcript-editor/shared/mS065fQZnxM-bqLPKA83IY9PR1UR4MmdD4I1qPAhjOBSIM63PhJnFJ25iuvg3L6m9bteJhjU9jIsecdDkRYUmhBcwVM?loadFrom=DocumentDeeplink&ts=444.44)):

And they send it through email or there is specific system?

Speaker 2 ([07:26](https://www.rev.com/transcript-editor/shared/lOwVDYVxIC_HQ83Gu1vBSG5BfR66ux-pW51x0Mw-FpVn1juOCZUJXp-7Q0v6yVPFSPOxSlaoyf1R8FPoD7_V9NbTsPA?loadFrom=DocumentDeeplink&ts=446.95)):

Yes. We have a communication email that is exactly mentioned. Your title and the other details for any action and plan coming from quality and patient safety level.

Speaker 1 ([07:39](https://www.rev.com/transcript-editor/shared/AL_uWeElTBs3q14sdHBGtie1U-oLLid79dXzKpOWX5JhcPzD9MLVdtqpnYQ1HCYk93CTy3httig-y75wVWgoMjR16NI?loadFrom=DocumentDeeplink&ts=459.8)):

Okay, excellent. And what about the DHA or is there a connection to inform them?

Speaker 2 ([07:45](https://www.rev.com/transcript-editor/shared/e7h58irX2cm5_v6vGKGG0PMqoeH-E1vGb26P8BbmO1jFPw0PFGcq6JTUZcfA6r0tf3vrO_CiqLtDnh2uwKv-WUgsh7M?loadFrom=DocumentDeeplink&ts=465.51)):

Yes. Now we have two type of medications here in Dubai, medication which is registered in UAE; we either notify the supplier about any adverse drug reaction happen or the company itself, for medications registered in the ministry, we report it to the MOH if its serious within 48 hours.

Speaker 1 ([08:12](https://www.rev.com/transcript-editor/shared/33tprjT-OaW88T1ph2kRDP3RKqAfmIkITrPpbtNTHe6lRecqrQrkmtPDbRo5c8flyqKl4GSLfZjabalInP4CycGzzq4?loadFrom=DocumentDeeplink&ts=492.14)):

Oh, so you informed the company when ADRs happened?

Speaker 2 ([08:15](https://www.rev.com/transcript-editor/shared/CBnhkLPN8ksSg2GUMx4Cr2vEcCU9PAuO8NgilfDX96T4xxcj1vkhmTz7siXbe6bTr9SHrtun4UqksM3vUEgIsxs7NXM?loadFrom=DocumentDeeplink&ts=495.14)):

Yes. They have to report it to the Ministry of Health or the Vital Health Authority. This is what we do. They have, because they have the quality and patient quality assurance and quality representative in their companies, in their branch in Dubai for the companies and for the non-registered product, this product is coming under responsibility of the hospital. So that means they don't have a registered supplier in Dubai In this condition, I signed a declaration letter that I will be responsible for any pharmacovigilance issue to be reported to them watch in case if anything happened with no responsibility for them. Because it's not reviewed by them, it's coming directly to them.

Speaker 1 ([09:07](https://www.rev.com/transcript-editor/shared/zbVznPqOxPkMveB5GYN2gxFZixzbs74MRqwfpnJIiWOqXV1L7vRhLDqQ6ojho6mWK74eCfhmsr7S-pBk_EOJoAwEGa4?loadFrom=DocumentDeeplink&ts=547.54)):

Okay, so because it's not reviewed by them, it's coming directly to them.

Speaker 2 ([09:09](https://www.rev.com/transcript-editor/shared/NjstCw0XhIzRwhDvi7gjNIj8gbB9_zO-DjQ6clxnbZHyDCEP4pucaX8vWC0YDmd71ReLffZE68bHIbRSu4roIF60v8s?loadFrom=DocumentDeeplink&ts=549.08)):

Yes. I can show you an example of this declaration letter.

Speaker 1 ([09:16](https://www.rev.com/transcript-editor/shared/rjINxyN_vkx4cnyELnlME20Hs8gT5uTklxuVHTi0H9Rozil5uHVjq1RxVUpqHv_h99ehcV87fGA1OVgXdW6a66ifUlk?loadFrom=DocumentDeeplink&ts=556.38)):

This is for Any product that is not available in the country or not registered in the country?

Speaker 2 ([09:17](https://www.rev.com/transcript-editor/shared/P3NkpzKq090CP9g13eIe6aa_cc5BvYMHoe55m3Mt9oym8NOl_xwAPdICFPrqR_FHM7J_274FwBQbcy_A7kYemxUlMdw?loadFrom=DocumentDeeplink&ts=557.97)):

Yes, See, without responsibility of ministry of health, any quality issue to be reported this declaration issue under their supervise and responsible to work.

Speaker 1 ([09:35](https://www.rev.com/transcript-editor/shared/uWohlxqCXQuIuXNMgTJ_ZJyaRV3KVKCrkKStkJG0EMT1OhVqgMuNntnf-JKMd3zCqkQxUuNuvUR8TNsNdy_m2Wn7U5I?loadFrom=DocumentDeeplink&ts=575.4)):

Okay. So, if anything happened directly, you would report to MOH?

Speaker 2 ([09:43](https://www.rev.com/transcript-editor/shared/j_qjSXLRpnx5q9O8peLMVfDFqnaYYtfU42XAYGBGOaL269vbUpdLxSaSHr2kIWiL0lH80N9__su5v98jTrIgtvwtPSA?loadFrom=DocumentDeeplink&ts=583.99)):

Exactly. So MOH or DHA as we are in Dubai. The pharmacovigilance is only one system in the country.

Speaker 1 ([09:45](https://www.rev.com/transcript-editor/shared/ad_HH39Lnj45Io1yt0sbAd4uyFPEdN2rnz0oPfxMLbhhSyVyjhLEkNA4VgsMnjXe0i9ZyM3QfMAFBNj4RVpvLc94f7o?loadFrom=DocumentDeeplink&ts=585.17)):

Yes. Okay. So, this is like the system available for the reporting of the A DR in the hospital?

Speaker 2:

Yes.

Speaker 1:

Okay, so any pharmacist in the outpatient and the same for the inpatient?

Speaker 2 ([10:04](https://www.rev.com/transcript-editor/shared/CQFp3RZhiAxdoMqvoJdnfnzf_MZAh5R4cn-NFyGM7Ax5p4fQzbi9k0C0VdP5VdiWpDu6GR_rUenW8Q8w79SOjhUmMFE?loadFrom=DocumentDeeplink&ts=604.29)):

Yes, for the inpatient, also

Speaker 1:

Okay, so for the inpatients, who do the reporting nurses or clinical pharmacists?

Speaker 2 ([10:04](https://www.rev.com/transcript-editor/shared/CQFp3RZhiAxdoMqvoJdnfnzf_MZAh5R4cn-NFyGM7Ax5p4fQzbi9k0C0VdP5VdiWpDu6GR_rUenW8Q8w79SOjhUmMFE?loadFrom=DocumentDeeplink&ts=604.29)):

Like a general role, whoever observe or receive an information that there is ADR has to report it. Okay. Example, patient develop anaphylaxis of a drug, develop any allergy, whoever observe it to report it, the investigation is coming in the next system.

Speaker 1 ([10:24](https://www.rev.com/transcript-editor/shared/njGLHUiAi16rY07xDrUOxZMgn7PTHxWdVBlL1huELgc0v5x_faEGRkvneYz6w4XBR5VLnoCShsude_P4H1lZTsejDcg?loadFrom=DocumentDeeplink&ts=624.75)):

And also it'll be done again with you.

Speaker 2 ([10:27](https://www.rev.com/transcript-editor/shared/AJLFVyPxwIfg4oziYQ3mFzhuNjYjIufL8dlSmyaibsqqxaiVTfhINpH1fgCpR5fxTi6qDB2ehmlfIat32n09vckOjfI?loadFrom=DocumentDeeplink&ts=627.4)):

Exactly

Speaker 1 ([10:27](https://www.rev.com/transcript-editor/shared/GWVOhPRyCoV4lHckHURsibSk-9FnKCf1ln6jPNMQvy00DhksZZR1InfJvf8VXdakVX7KW9f4-hIIAAcjJO8qwN17bbo?loadFrom=DocumentDeeplink&ts=627.68)):

Is this the system at your hospital?

Speaker 2 ([10:28](https://www.rev.com/transcript-editor/shared/pC66IY_SboywuM6Vn1VAOZ2J6YeHP1CTxVwMHCaJMUeeNMhdI5YEw8TjzGOjJ2QoYS88cCNcgnPfGjoaCy8lNoy2giA?loadFrom=DocumentDeeplink&ts=628.14)):

So the system structured anything related to medication, it'll come to me even if it's reported by pharmacist or reported by nurse or physician. So I have to put an input related to medications.

Speaker 1 ([10:40](https://www.rev.com/transcript-editor/shared/gu5grr_tdmjDAlpTnTsHDZnvUPZFUsEJhC6gpG6N3r3dPtCV991PjMBITwX4bn3J5cd2HHhjh3aOsc8MOHYR5aHuLRs?loadFrom=DocumentDeeplink&ts=640.47)):

Nice. Then it'll go to the quality to evaluate it?

Speaker 2:

Exactly.

Speaker 1:

And they decide or you took the decision ?

Speaker 2 ([10:48](https://www.rev.com/transcript-editor/shared/J3ERQn1LuSTs2uh87nNyGIa4Lw7sInIhAqzEt2Vjn13AxJcFyd2HC08NxUPSGOGlW8DKU9Aymzz0MzLpDoRXPg6IMWI?loadFrom=DocumentDeeplink&ts=648.33)):

Then again, they Have to review my plan and approve it or revise it in case it needs further information.

Speaker 1 ([10:55](https://www.rev.com/transcript-editor/shared/sXBhlCz-VI65SLWWHoaER-Zuwqsd1-d0EqaF5RrCu5r0Gkn7Q0Om4jonQUmVzokmSypSmM6Q9AqirQyrYNF5IlZ_ljk?loadFrom=DocumentDeeplink&ts=655.26)):

Okay. And you can meet with them again and to do the Decision?

Speaker 2 ([10:58](https://www.rev.com/transcript-editor/shared/sgHmUeIo30i7acl4fo64zxvByKUZeeTlhOMoO65SA6s_xzB4DBuEzgBSr69fj7D9-bTo6-3M4GsM9oHVT3wBbA1qTXI?loadFrom=DocumentDeeplink&ts=658.21)):

Exactly.

Speaker 1 ([10:58](https://www.rev.com/transcript-editor/shared/WPWphntEQC_M1BgC1yUu-ujE6j7QxZGiNJGZxoDGwLV-xeYj24BhwolDNNTI3w6PnMPQOufMogGpOq7vBspa6edwjg4?loadFrom=DocumentDeeplink&ts=658.77)):

Okay, nice. So did you take before any courses or training related to pharmacovigilance, medication error, drug safety?

Speaker 2 ([11:08](https://www.rev.com/transcript-editor/shared/e_iZGEgFxLvuYplueUXBl4hcPuZAmOMTuYCHirYmsaLbOa635lVhljQCoBK2dmM81dFGnbjuwph0O6w3XVoW394CbjM?loadFrom=DocumentDeeplink&ts=668.91)):

Yes. Because here we have two standards. First one which is American Society of Health Pharmacy, A SHP. The second one is the GCI and the GCI also the SMP, like what is the definition? How to report.

Speaker 1 ([11:27](https://www.rev.com/transcript-editor/shared/3-A4eplj73-wDkXdYUi30khHQHg9Tl4DOJO1EwxpGnN8acyFkz6SVobfvd1hYdzMjBatD2iR6SSJ5xfrlvYpojp8ZzE?loadFrom=DocumentDeeplink&ts=687)):

So both require that you have to take a course training courses. So you took Yes. Okay. So it was a training or training you attended or courses you Said?

Speaker 2 ([11:38](https://www.rev.com/transcript-editor/shared/yZvCrx2Iomf-1lAEXzMATV7rfAzswxhVdKFTXXTuqJyfk8IUtBtcTl_ivkwNcleCH45JBjx0tCYMbfPPV1NZ6r8rYyk?loadFrom=DocumentDeeplink&ts=698.37)):

In our previous hospital it is mandatory training. Training. Okay. Here we make it as part of the orientation or the pharmacist. Or the clinical pharmacist as well.

Speaker 1 ([11:52](https://www.rev.com/transcript-editor/shared/eTkxWJ27kOedc1UtaMxWw1l0Zt257kiudpZqx4Qft-TuHQcbgCCczgsfKp83lBdzef4313e2RrPgKXXsvupFoi_rh4U?loadFrom=DocumentDeeplink&ts=712.02)):

So, for any pharmacist, he's joining you here. First you have to give him an orientation about the medications.

Speaker 2 ([11:57](https://www.rev.com/transcript-editor/shared/ltPf3QtAVZTyN4R0bSsKchoNKrlrp4UMQ_3yfQGPj5mNsYuQlmRbP1ZNuuLjh4_qZtk35y0gz9a2HB7tUGxKcdJ4_jM?loadFrom=DocumentDeeplink&ts=717.97)):

Yes. Orientation about exactly medication safety measures that we have here in hospital And reporting of ADRs.

Speaker 1 ([12:03](https://www.rev.com/transcript-editor/shared/fxJECZRzv_N32Hr_8frWQjV2UsRSrirKrqRfJw3TD-NZiqc7vBCX0Yi63sJCnp1lDV5ehL-E_-EgKcH4dCro54QdnoI?loadFrom=DocumentDeeplink&ts=723.03)):

So, you are encouraging your staff about the reporting?

Speaker 2 ([12:03](https://www.rev.com/transcript-editor/shared/fxJECZRzv_N32Hr_8frWQjV2UsRSrirKrqRfJw3TD-NZiqc7vBCX0Yi63sJCnp1lDV5ehL-E_-EgKcH4dCro54QdnoI?loadFrom=DocumentDeeplink&ts=723.03)):

Yes.

Speaker 1 ([12:03](https://www.rev.com/transcript-editor/shared/fxJECZRzv_N32Hr_8frWQjV2UsRSrirKrqRfJw3TD-NZiqc7vBCX0Yi63sJCnp1lDV5ehL-E_-EgKcH4dCro54QdnoI?loadFrom=DocumentDeeplink&ts=723.03)):

Okay. So what are the ways of encouraging them to report, especially let's say for the outpatient who are just coming?

Speaker 2 ([12:13](https://www.rev.com/transcript-editor/shared/D5RPn3fpU_79ZK7jHAnGyi8QGBMSFqHgF6izQcTDGEuEFkgDSrk2Zt4u_WC3zBaEb1VHGOQF-L5KCTfuQZ6JUT2rBlE?loadFrom=DocumentDeeplink&ts=733.54)):

We have different ways of encouraging ADRs reporting, one way we follow the non blame system. Non blame system means that our concern is the system, not the one who make the issue. So I received the report for people who has observed drug reaction or who has make the medication error. Yeah. They report by themselves then I did the following and the patient end up with the following.

Speaker 1 ([12:41](https://www.rev.com/transcript-editor/shared/WO1CZ3SOaPgQfRw3KoX2pyAjmjeMfSknjFbuBGShzgK2TweiuGgIMhzQrmcEG9M-eW-5cvq1LGkRfOdb9j99NVZMc5I?loadFrom=DocumentDeeplink&ts=761.14)):

Okay. This is by physician, right?

Speaker 2 ([12:43](https://www.rev.com/transcript-editor/shared/09RD08RNkAiGLoCK323NeF-P8VlQO7o4LIzy2w5d5hmiM1QG1TM9fQVVROJK2mcj-ELFKfDlhKuPnho6sJZeCLDo-KY?loadFrom=DocumentDeeplink&ts=763.15)):

By pharmacist as well?

Speaker 1 ([12:44](https://www.rev.com/transcript-editor/shared/B6E2k9k6_OIHyRWAPZO5uutdCBoa7OQAaXnMjHPeBL5SvmNxPsZzi35GIyISdjFOHENW-xIcJRD_KogRq-p415Xu-7k?loadFrom=DocumentDeeplink&ts=764.45)):

A pharmacist also they can?

Speaker 2 ([12:45](https://www.rev.com/transcript-editor/shared/rPZA-E16zZSaZGbOzTsAxSZ7vUQg-TYNH674Kt1h6oXN0QPETNOgHkELocY8_fgTH7scPOynKRY5EQ0BwvuPH8fCYO8?loadFrom=DocumentDeeplink&ts=765.74)):

Yes. They can dismiss. Yeah. Like for example, if he dispense not high dose like wrong drug, this is an error and patient develop a reaction related to this drug? Yes. We have an events before that reported by the pharmacist who dispense it, who make the dispensing error.

Speaker 1 ([13:05](https://www.rev.com/transcript-editor/shared/Bw5mIfWl73Is9mjWUuXVu7KAjfbkC9hmDBwLBZyObXj4G_udKu5N94f-Cd8Aq4XlUsxpffSp3d-wv2RrdhgVndjgBG8?loadFrom=DocumentDeeplink&ts=785.43)):

Okay. So what are the most type of ADR happened before from your experience? Related to what?

Speaker 2 ([13:14](https://www.rev.com/transcript-editor/shared/AUHUMDAcfudI7sNhg6y6Ghh4eZPggNq9xIsn7MBOIycYy16h71oL0GiHzIu8c7rHetoLm8_X82nhBV7h_UsQ8C-ShVU?loadFrom=DocumentDeeplink&ts=794.86)):

The most common related to the drug itself, like part of the mechanism fraction, which cannot be prevented. Example, hypoglycemic agents for patient developed hypoglycemia, hypotensive agent patient develop hypotension. So this one cannot be prevented. It's part of the effect of the drug itself or patient develop dehydration after receiving multiple doses of diuretic and the other type of events, which is the medication error, prescribing error we have even with availability of EMR, still we have a risk of prescribing error. Dispensing errors happen also before administration errors as well. This is, we are talking about the medication error itself.

Speaker 1 ([14:00](https://www.rev.com/transcript-editor/shared/0dE8kpbkdPuOTueVHsPrAIOKh3gr5KG2lHXV3rR5ZyAEHF8Bd2-LxbqyQBma7a4LLs99in7TbnUkr2SsLSAXRdbXe-0?loadFrom=DocumentDeeplink&ts=840.94)):

Administration error. Like what?

Speaker 2 ([14:02](https://www.rev.com/transcript-editor/shared/mNdqJp2ChI4RXNCPksPCRxysOBWxwbpDaR0IxLMSyK1ex6m0srLN7tz9n6SlWofozaNhMF-PuPD4LoUz6j59QVtbQ2o?loadFrom=DocumentDeeplink&ts=842.9)):

Giving medicine faster than the recommended infusion

Speaker 1 ([14:09](https://www.rev.com/transcript-editor/shared/HAela9E2rxnFZgYn9GVIPGFHTWEz6Hsewk4aadeooAvyguPFLYawWg4XQjB-3K4kUB2zOyIjT7HT55jL9EZWhd_BaYo?loadFrom=DocumentDeeplink&ts=849.09)):

Related to this is inpatient?

Speaker 2 ([14:11](https://www.rev.com/transcript-editor/shared/6Phyn93dm4azP_jTljvc32CaBzvHoPzAMIGrYUprrnbee8CFwSho6DdsoBfFmRqYHESorZ8wF-A8Xybz8MrBLldF8po?loadFrom=DocumentDeeplink&ts=851.03)):

Yes. And end up with reaction example, the drug should be given over one hour and given over 30 minutes should be given over 16 hours giving faster than that biological agent to start gradually while given faster than recommended.

Speaker 1 ([14:27](https://www.rev.com/transcript-editor/shared/SeMnLfEDA_JUO31fag_v9Yqzhf3E9V0DD5CLf9t9mDqEFCYlQx5fIZiCAV19z7KHnr8hYsSCP0TFSUMP2iYBV0N2IPA?loadFrom=DocumentDeeplink&ts=867.6)):

Nice. Okay.

Speaker 2 ([14:28](https://www.rev.com/transcript-editor/shared/y1RxwZ1Ru2A1cGuRRNZk1Ym_nP9TPevhsHRbCiyGjCssgmuZQiFj-OAldc5B-VXfHknTDc2wmJegIkUbapU0fSgC5DY?loadFrom=DocumentDeeplink&ts=868.66)):

Okay. So from our side what we do, we encourage the one who will make it to report and we put the risk mitigation plan.

Speaker 1 ([14:36](https://www.rev.com/transcript-editor/shared/xu2tAMChfUba4XoPNotEeaPNAM9_n6Rj1QF2DI4gGHbyAEwyC3v9bkJGtIUHV7YrKJTWPnwAH-ODaOJPmv0GNkHBaMM?loadFrom=DocumentDeeplink&ts=876.88)):

Okay,

Speaker 2 ([14:37](https://www.rev.com/transcript-editor/shared/pvGT_FP63CttK9UKRQqZHQUk5sPlwX_hR9puxogeNAGgBWRogg3FKsO0Tqy07_e-R1HDc6CNO7LJAnRYxK9lVQAn654?loadFrom=DocumentDeeplink&ts=877.45)):

Excellent. Since it is not preventable, we have the mitigation plan for non-preventable reactions.

Speaker 1 ([14:44](https://www.rev.com/transcript-editor/shared/GdSkDw4YjNex6gNFwdAOEyLxkOWuw1CWUeeYb49KiIMIuZMddo3PUg_3CEj6ZwHuJtWLv4bKRECNU3yGHb40fv53cEI?loadFrom=DocumentDeeplink&ts=884.98)):

Okay. So here the question now, when you are reporting to the ministry of health MOH, is there there a specific period that you need to report within 24 hours, 15 days,

Speaker 2 ([14:56](https://www.rev.com/transcript-editor/shared/QNzzWpJM-jsSYeguebj3ZiTTiDaHM4dW4ab5wKizFxswPcoXhp6P8sRruCh3z_cXSrR6TQeIyHpbglN7BK0NnLPYcKA?loadFrom=DocumentDeeplink&ts=896.21)):

10 days up to my knowledge, the serious issue to be reported. Okay. Especially if it is a new drug within 24 hours.

Speaker 1 ([15:03](https://www.rev.com/transcript-editor/shared/by9GXjd117Gsc-cHugiW_RzMdxTVb4jjg6lLjc9RVwAyQ72DyAGCGyKhPE4WSG3io09DfRFVYhOZ6jUFuquODCvgKrQ?loadFrom=DocumentDeeplink&ts=903.24)):

Serious only adverse drug action within 24 hours?

Speaker 2 ([15:07](https://www.rev.com/transcript-editor/shared/HymUG-JDkWAg1mnxPUTG4Q9SF8H2AGU6_6wiAdFhqRQM1JgCfAJ9gX4vp80k_fgrEXvxQY4X26A0Dt4BgJ8edJmjk4E?loadFrom=DocumentDeeplink&ts=907.83)):

Yes.

Speaker 1 ([15:08](https://www.rev.com/transcript-editor/shared/fuZmvZuC0yR6lAsYrfY1kf0klFVpPldWlJqAoKjRsYc7nag4iwCC4hqJsFAcWsew4vSUsXgAzzyYpAs2xRg2goh0Hx0?loadFrom=DocumentDeeplink&ts=908.99)):

Okay. And if it is not serious normal, you are not

Speaker 2 ([15:11](https://www.rev.com/transcript-editor/shared/ZkYLIjM-xALmPb_1o7GrB1FGnfixpMJF0AC46_J_KZ0yOnSh6GtLVXe-nV0AF6JrJEFA3nB_9-ruHSgpT0nRszMqBmU?loadFrom=DocumentDeeplink&ts=911.57)):

Usually internally Yes. You don't report it? Yeah, we report it internally.

Speaker 1 ([15:16](https://www.rev.com/transcript-editor/shared/Zl9twBf2jg7OYKKlayZ0Cu2q6WWrFAUW4BiqtsSBIk4DtkdLYh5opnCbcxIcOu9PH5MjBEjowFlqbQIlnTFz_IMkUD0?loadFrom=DocumentDeeplink&ts=916.05)):

Okay. But here, even if it is minor, they have to report it. Yes. You are encouraging.

Speaker 2 ([15:23](https://www.rev.com/transcript-editor/shared/rv6RMA3oXgGshGucwkLc6WxCvLoWc9uvoRolPh7nENFfuohk9inOINuf9sU0lUIkV4hAY4w8qItUbO5IruEAGY1sG5k?loadFrom=DocumentDeeplink&ts=923)):

Yes. Internally we did the reporting, everything should be reported just for a purpose of improvement, but not for the ministry of health.

Speaker 1 ([15:30](https://www.rev.com/transcript-editor/shared/S40RzOvW1FQj7Z1vrIFetoEtOq52EYVaISAKdqfSHqp8s_3bWYkV94l2kqZ9luDdYseBzgZfY99iL3nBUaG8ahW5XNQ?loadFrom=DocumentDeeplink&ts=930.72)):

Do the pharmacist, they started to ask the patient if the patient is not talking, they are always in a hurry just to take the medication and leave. Do they start it by themselves to discover if there's any ADR or side effect for the medication With the clinical pharmacist?

Speaker 2 ([15:44](https://www.rev.com/transcript-editor/shared/VbNMsqEvVYbddDEI1bMvCKNnxyqIVbAoaGt9-KgbwN8KArq1fDejXynI5QaqPkULeOOSXLXq4LMtqPEMI_ccsbbsVgs?loadFrom=DocumentDeeplink&ts=944.85)):

Yes, because we do patient education upon discharge, upon admission.

Speaker 1 ([15:52](https://www.rev.com/transcript-editor/shared/By8fdqTOFjpFrTjdmsz2Va_Quw6VADmqtvyvvbEluI6ZWsGI0bhrVknH35lVKZgNlb2ghetH0NkdgxPQEd2U9nlTh-A?loadFrom=DocumentDeeplink&ts=952.24)):

So mainly for the inpatient, right? Yes

Speaker 2 ([15:54](https://www.rev.com/transcript-editor/shared/a7x0VaP2apDZ1I9_cIjb3GF8rK3JVyFipT29f7SK2yJ2Qpcm1K55ILx6OLK-dk9phPxlLk58m3GV3gEk0bAt36tb_xU?loadFrom=DocumentDeeplink&ts=954.86)):

For the outpatient usually they discuss it directly with physician and initiative by pharmacist is not frequent. It is what started by physician, but patients call us to follow up like he notice rash, right? Yes. We receive an email from contact center, somebody interested to call pharmacy. We call him back to discover

Speaker 1 ([16:20](https://www.rev.com/transcript-editor/shared/c3h7WQqvRqW5SBUfM5uNgYlzcbBY_Oi0-jA8RbhVNGDldkN7pHZm2yfLezvxZWTUWkBt_045ZJVim5oUw2D_kDo3I1k?loadFrom=DocumentDeeplink&ts=980.12)):

What his, yeah.

Speaker 2 ([16:20](https://www.rev.com/transcript-editor/shared/x6R9_TT_fkEBmU3EewnbcmhQUSr1eIQfWIgT06LHE_0hVxcUdoEIbRPT88tXFgx2x884hZWI9k142wqxAK45mwhLflM?loadFrom=DocumentDeeplink&ts=980.33)):

To discuss what he has and sometimes yes, we found that he develops an adverse drug reaction.

Speaker 1 ([16:26](https://www.rev.com/transcript-editor/shared/jjObicNeWr0N_nY2WKzWjwv93UJ7Z84eHPnt-ujF3AKpVkyH2nQpvlvRZmNpkyzIcDGCF7mJtAKqsZ8qIztvXvyMPZs?loadFrom=DocumentDeeplink&ts=986.66)):

Okay. And what’s the action?. You ask him to come back to the hospital?

Speaker 2 ([16:30](https://www.rev.com/transcript-editor/shared/vMQL-DBAcm5MKPeaE1qVQYWJgtvDt8sDVrzrIOvsW_1eJlGvnKO3BjhyNLzEH4GOXl79UkpHccU7KQWmGWbd1Olf9Bk?loadFrom=DocumentDeeplink&ts=990.84)):

It depends if it's something common and does not affect a quality of care, we give the advice. If needs medical referral, we refer it to the physician.

Speaker 1 ([16:43](https://www.rev.com/transcript-editor/shared/z1o0dHSySR5kx5HtZ2Qste7RZnSSDSZw2IsyTGB53DTWTjv1-VWeSewTocquxJOVI1H9r7E0lTYWfdm-V_MHfMQhNQY?loadFrom=DocumentDeeplink&ts=1003.26)):

Excellent. Okay. So what you think from your experience as Dr, how we can improve or increase the ADR reporting among other hospitals in UAE in general, and Dubai especially first of you manager here of the Pharmacy?

Speaker 2 ([17:00](https://www.rev.com/transcript-editor/shared/bjkZxa9KINvYMSVq53zmYEAu3ahUE9BCgFdZHDRt3KOOanAnitS6YRFjmRJ5idLHls58ZOa-oJacDP5usAdYykoXOaA?loadFrom=DocumentDeeplink&ts=1020.42)):

Yeah, first of all is the awareness because I notice most of people believes error has to be reported only or something unexpected. While we need to know all reactions is related to the drug. So the first all is the awareness, the meaning of reporting, the importance of reporting. And then I'm non blaming, like this is really not related to take an action against the person who report or the person who observe the reports rather than improvement of the service. The other thing is the measures that will be taken, people has to see the result of their reporting. Example, if you have many reports does not mean the service get worse. That means maybe rate is improved, rating of reporting is improved. And then after that reevaluate the quality of these reports. It's showing that our plan does any improvement or not.

Speaker 1 ([18:05](https://www.rev.com/transcript-editor/shared/FFrJ6L0nL2ffRID0eIClNNxBdkL6QJT-zHKvtHuCNqUsKXxNDrnXNuo3Q_5B5hf4LYQhYu4W7BIXep7a6GtRHYu__Ik?loadFrom=DocumentDeeplink&ts=1085.36)):

Okay. So if I need to ask you how many the average number of ADR reports have for the last year?

Speaker 2 ([18:13](https://www.rev.com/transcript-editor/shared/kynfBqlAsNcZJTp6sYZchIsIwg4XKfG_3Fotzd6Dt8FRpP5kaadMxjhbFQuOuQVfDm4WZ6uV_tiH8LvlGbbk-E92dsg?loadFrom=DocumentDeeplink&ts=1093.71)):

Myself, I reviewed per month, maybe 10 to 20. Oh, okay. Okay. 50% reported by pharmacist, other 50% reported by physician, other healthcare provider nurses, et cetera.

Speaker 1 ([18:32](https://www.rev.com/transcript-editor/shared/uRjByfuaQjUxCGJlJ6nIJvjKNB3-P1fyZXgeAJQqK2_cic21idEoL59yd4HVqRNtGrk9X-gsYl72tfiO4E_RUOPb4wQ?loadFrom=DocumentDeeplink&ts=1112.22)):

Okay. So Dr. Omar, as you said, it is like 10 or 20 monthly. So what are the types of these ADR reports?

Speaker 2 ([18:42](https://www.rev.com/transcript-editor/shared/ad-isw8V7MMEP2IMae6OyBlnjufdFTdhmXteaH-YQb2Rn85_oJ1Kddxl7lNSH-Hf1SVOCOze_7C-ZRRzm9tw959QKtM?loadFrom=DocumentDeeplink&ts=1122.15)):

Majorities are here. Okay. It is equally distributed between prescribing administration and dispensing. Minors are related to the drug itself or reaction related to the drug, but majority are errors are preventable.

Speaker 1 ([19:07](https://www.rev.com/transcript-editor/shared/i2D8FGbtGqQFbyIam_HtC4zPyDQfMBrnH2rMtiHA9CGLMxI3UGrIu7V9F6xjCDDej04gmXFE7FgYm9fYQeRC4dCpLcI?loadFrom=DocumentDeeplink&ts=1147.79)):

Excellent, preventable. Okay. So is there a regular training or awareness for you to staff here on the monthly basis or

Speaker 2 ([19:22](https://www.rev.com/transcript-editor/shared/VHc71YwfX3pp163C1nUk8Ss9VbWkkSQfF3wamh4lKwDorA4CBvGCd8kDpBXeJd5O6THWU8OD4zo4ylLFWbSjr9TMHJk?loadFrom=DocumentDeeplink&ts=1162.02)):

Yes. What we do, we have a departmental meeting every month for the pharmacy. It has a standard agenda. One of the agenda, which we start with it is the last month events either reported by pharmacist or reported by other department. All medication related issue in terms of the details of the events and the action plan, it is done in monthly basis.

Speaker 1 ([19:48](https://www.rev.com/transcript-editor/shared/EjL2q_G--0-akLJJKZPPn_GMPSHNX45jnx3v4Nt2dMjSaBqCO7AeBL5ZjjTRSH785wt-5Exf9WQtJk_KsiqXioQ-ibo?loadFrom=DocumentDeeplink&ts=1188.87)):

Nice. Okay. Thank you so much Dr. Omar, for your time and for all the information you give to us. Thank you so Much.

Speaker 2 ([19:57](https://www.rev.com/transcript-editor/shared/U2gagryDItRHpdCU46Pi62BttCetI3z-YZow0-LBOVUPJ-4VoTBaDj3TMs4PjC6cTov_1u2AYTEkFmX7AUyGSkKVNAE?loadFrom=DocumentDeeplink&ts=1197.07)):

Thank you.
