# Supplementary material for: A qualitative study to investigate pharmacovigilance systems in Dubai hospitals
Source: PLoS One. 2025 Sep 10;20(9):e0331929. doi: 10.1371/journal.pone.0331929 (PMC12422479; doi:10.1371/journal.pone.0331929)
Supplement: S1 File — (ZIP) [file pone.0331929.s001.zip › M5.docx]

Speaker 1: Good morning Dr. and thank you for joining this meeting. Hope you're doing well. Thank you very much.

Speaker 2: Thank you. Good morning and I hope good luck for this meeting.

Speaker 1: Okay, so as I told you earlier, this is a qualitative study to explore the PV system and the existing PV system in different hospitals in Dubai

Speaker 2: You mean pharmacovigilance, right?

Speaker 1: Yes, the pharmacovigilance, exactly the constant form. I already shared it with you earlier and you sent it for me. Thankfully

Speaker 2: I read it and I signed it and sent it back to you.

Speaker 1: Yeah, thank you so much. So this face-to-face interview will be recorded and later on will be shared with my supervisors, co supervisors and also for publication. It'll be used for the publication. Do you have any concern about it?

Speaker 2: This is part of your study, right? PhD study?

Speaker 1: Yeah, part of my PhD.

Speaker 2: Good luck with your PhD and I hope you will have good results at the end.

Speaker 1: Okay, thank you so much. So first, can you please introduce yourself, your experience, years of experience, your ranking and your qualification.

Speaker 2: Thank you. My name is ,,,,,,. My professional backgroundis: I am basically pharmacist. I studied bachelor pharmacy. I graduated from Suna University back 2000. I work in different sector of pharmacy. Initially I work in a pharmaceutical company for sales and promotion in Abu Dhabi Uua for around two years. Then I joined a private pharmacy for another two, three years in a private sector in Dubai. After that I joined, I'm moving from section to 16. I moved to hospital pharmacy and there I work in Dubai Hospital as a pharmacist. Initially in outpatient for a number of years. Then shifted to inpatient and I focus in on the part of my job there in TB in preparation and then chemotherapy preparation. And the last four years there I was working as super user pharmacist for ePharmacy training where I start my master from Aman University and we were college at that time.

Speaker 2: We are the first class of master in Ajman University. After that I did another master in public administration and I did four different American board in different specialty including oncology, pharmacotherapy, infectious gene and ICU. And then I moved to another hospital, the military hospital where I shift my practice to clinical pharmacy practice back 2015. There I practice in ICU in medical ward, surgical ward, cardiology ward or ward. I work each ward at different time. I finished also my PhD in healthcare management from Hamda Hamed Martin University back 2021 and now I joined new job in ,,,,,,,,, as a head of pharmacy almost 10 months I am in ADA center as a head of pharmacy and that's it anymore you want

Speaker 1: Long experience.

Speaker 2: So it's more than 23 years of experience in the field.

Speaker 1: Okay. So now if I want to ask you about the pharmacovigilance in your institution. So is there any pharmacovigilance center or any person assigned with the responsibility for monitoring ADR reporting in your working place?

Speaker 2: Yeah, actually my working place, we are relatively small center and our pharmacy ranging from three to four pharmacist including the head of pharmacy. So that's why there is no person specifically done for vigilance. Generally speaking a DR could be reported by anyone who witnessed any healthcare provider who witnessed the A DR or the adverse reaction. So is it a nurse, is it physician or pharmacist who will witness and from the first point that a DR, he's the one who's reporting and the report will go to head of pharmacy for evaluation that report and then it'll go to the committee pharmacy, BTC and also if there's something really new things not happen before we report it also to the authorities.

Speaker 1: Yeah, we will talk about this point more in details later on. So among you the pharmacists in the pharmacy, you don't have one specialized to be responsible about the PV?

Speaker 2: No, we don't have one specialized, we don't have center even or one person who's specialized for this task.

Speaker 1: Okay. So is there a clear meditate, organizational structure, rules and responsibilities for the pharmacovigilance in your institution?

Speaker 2: We have policy, we merged it ADR policy and medication error policy in one policy and in that policy we set the responsibility who should do what, so who should report it and the form to be filled for reporting that and the timeline and then what are the stepwise to be taken in this.

Speaker 1: And this is for all healthcare providers in the hospital not only for the pharmacist?

Speaker 2: Yeah, all healthcare provider including nurse, pharmacist or physician, either medical or psychiatry.

Speaker 1: Okay. Is there any an annual budget allocated for the PV activities in your hospital?

Speaker 2: I don't think it need a budget per year. Encourage it scenario especially we are small center as I mentioned before, it's part of the pharmacy or yeah, part of the pharmacy daily job day-to-day job.

Speaker 1: Okay. So what is your experience with the A DR reporting?

Speaker 2: I have quite experience in all this hospital I mentioned. So in Dubai Hospital for example when we were in Dubai Hospital there was a system, it's called Aman System and where we used to report any incident and one of them is the ADR report and it's online and it'll go to all head of concern department and if they have any comment they will add that in the loop and everybody will be aware about that. ADR and in Zayid hospital there was a person assigned when I was there, if there's ADR it'll go to one clinical pharmacist who's always responsible to follow that one and do more analysis and deal with the quality head of department to see what is the next step if we need any more action or if the medication, for example one time in anesthesia the airport they changed the brand and they said the anesthesia complained that it's not causing sedition this medication to the patient compared with the previous brand. So what's the process that time they said we need at least two or report to support this one and they report two cases and then they investigate and they decide for example to change that brand.

Speaker 2: And recently in this one patient who were treated for addiction, he used one drug is called buprenorphine, which is morphine anti partial agonist used to treat as a maintenance therapy for opioid dependent patient. This patient initially he will get this medication as subular, which is buprenorphine plus suboxone under the tongue and he did not develop any side effect. After that he will be shifted to the injection and once he shifted to the injection he start rash, then he got the 16 mg, he report that he could rash, increase the dose also 24 and he could antihistamine but the rash continued the same, it did not go higher. Then the doctor called me, she said while the patient on 24 she said patient want to increase the dose 2 32 can I increase the dose? Then after discussion we said listen, it look like it's maybe not necessary to be from the drug itself because he took the same substance under the tongue but it could be maybe from the latex available in the injection itself and less likely it's dose related. So they increase what we advise to transfer patient to center where the allergy center where they will discover what's the cause of allergy because the patient insists to continue on the dose although there is a rush and I told her we need you to fill the A DR form and the company we'll send the a DR form also. But when we inform the company, the company also they were interested to have as a pharmac to report it to the mother company. So this is an example I want to highlight recently I fish.

Speaker 1: So from your experience, what did you see the types of ADRs are must reported in your current hospital or a previous hospital?

Speaker 2: Usually we'll look to the side effect of the adverse effect happen to the patient. If it is in the leaflet of the drug, is it part of the common adverse effect and it's already well known so such ADR I don't think we need to report because it is well known side effect of such a drug. But the thing which is less likely to happen and that will be discovered during the drug available for large scale, especially for the new drug, if there is a new drug in the market which is not yet studied only on a small scale and now when we go to the large scale there is something which were rare, it can appear more with more patient. That is very important to report it because such report could stop medication from going more in the market and we witness such problem like insulin. I remember nebulizing insulin when it was introduced to UA market it was widely used and suddenly it was withdrawn because of respiratory side effect that time, I dunno now if it came back to the market but since then it's not back to Dubai authority as I know.

Speaker 1: Okay and from your experience who did you see that they were reporting more ADR physician, nurses, pharmacists, the patient themselves or there was not from any one of them?

Speaker 2: Yeah see to be honest with you, everybody's busy and time is limited and if there is no follow-up there is many ADR is missed because of this time limited and people, yeah,

Speaker 1: We'll talk about the obstacles later on in more details.

Speaker 2: So what I mean to say if you want there should be some part of the hospital responsible to follow this one if you follow of course personally I think physicians should be the first one who report the case because they are in touch with the patient more because if you are in the pharmacy and just about the they without or if there's a clinical pharmacist who's in the ward who's really following his base and there's interaction between the patient on daily base and monitoring of the drug after giving the drug. So somebody who's really interested to do that, he will be committed to report that one because he follow what happened on daily basis.

Speaker 1: Okay and from your experience did you feel that the ADRs happened more in one system or one department more than the other departments? Like from pediatric more surgery cardiology or there were not any specific? No,

Speaker 2: It's not specifically depend on the himself on the disease that we are treating on the volume of patient hospital dealing with. Of course the more volume of patient, the more A DR, the more type of medication. Some hospital they have more than 1,500 items in there. So if they have more items in there formerly the risk of having ADR is definitely higher. I mean specialized hospital who have more tertiary hospital who have all specialty has the risk to or have the chance to report more a DR convert with small centers.

Speaker 1: Okay. Now could you please provide us with a detailed explanation of your hospital's ADR reporting procedure in your current hospital?

Speaker 2: Yeah, in my current hospital, once the patient complain either himself proactively or through investigation with the physician round or even to the nurse that one who witnessed that A DR should fill form ADR form and send it to the head of pharmacy where the head of pharmacy and his team will review that one and see its evidence and then go to the next level which is reporting to the authority if needed and also to the BTC for they are meeting every three months or monthly and if needed also. So to take action if needed action for example if this is really serious and we need to withdraw this drug or it's just a side effect commonly happen. I have one example also I remember we have one in our center, we are using many drugs for sedation. One of these drug is Trazodone, so Trazodone plus quel plus other drugs.

Speaker 2: So one patient he complained that he have the prior patient from this drug and when the doctor, he report the A DR. Then when he report the A DRI went to him ary and discussed with him for he said actually I witnessed this with two previous patient also but I did not report the A DR. Then I mentioned that in my comment and I review, I did a review for the literature and I found it's not commonly happen but if it happened it's really a problem and we have alternative in our hospital for other sedative medication like what we said Cirque. So the recommendation is to reduce the use of this medication in male and keep it for female because this side effect is more commonly in meal and really the consumption of the drug is went down dramatically after this decision. And especially we have, there was a consultant joined recently who support the same thing. He said, yeah, it's not common, it's not common but if it happened really it's a big problem so better to avoid it. And still we have small people of patients using this drug but the number is reduced dramatically based on this a DR report.

Speaker 1: So do you have a special person or the committee who do causality assessment for any ADRs have been at the hospital or the clinical pharmacist or you as the head of pharmacy are doing this?

Speaker 2: Yeah, it'll be reported first to the head of pharmacy and they will ask me to, but actually it'll be reported to the quality and then quality will refer it to me because with the form itself, when we fill the A DR form also we need to form an incident report about that. So the incident will go to the quality and that quality incident is the structure that if there's a people need to put their comment, they can forward it to them and one of them head of pharmacy and then I will send it to them again. And as a part of PTC we'll meet and as a committee we'll discuss and take a group decision or committee decision based on this case for example or that idea.

Speaker 1: So you do have your own system in the hospital for ADR reporting? Yes. Do you mind if you share with me the name of the system? Soft?

Speaker 2: No, we don't have a soft, we have a form.

Speaker 1: It's like a hard copy form or soft copy form

Speaker 2: Like A, B, D, F. We need to fill that pdf Form

Speaker 1: And send it. Then you send it by email?

Speaker 2: Send it by email, yes.

Speaker 1: Yeah. Okay. So is there a workflow of ADR reports to a national level? Are you do sending these reports and regular basis to the ministry of health or you just keep it for yourself at the hospital level?

Speaker 2: No, we are keeping it in the hospital level unless something is really,

Speaker 1: If it is so on series ADRs you are sending to that ministry?

Speaker 2: Yes. Or something really interested or new things is not discussed before.

Speaker 1: And what is the process after that? Do they reply back to you with the action that you need to take it or

Speaker 2: As I told you, I am new with my organization. I am just 10 months and so far we did not report anyone that the one I told you about Trazodone, it's known in the literature and it's a side effect, well known side effect of that drug and it's rarely and at the end of the day it's a clinical decision. So we decide we have alternatives so we decide to stop the using got or reduce it. So we don't believe there's an necessary to report everything. But if there is something like a new side effect which happened first time, definitely we'll report it to the MOH and we'll see what's the response. Definitely they have a system in place but we did not have this experience so far.

Speaker 1: So only the serious ADR you are sending for Ministry of Health, you're not using the online form provided by the ministry to fill these ADRs happen at the hospital.

Speaker 2: Actually I adopt now we have our own form before, but now I adopt DHA form as a form for reporting. So we'll report on the same form which is adopted by Dubai Health Authority.

Speaker 1: But you still use it at the hospital level? Yeah,

Speaker 2: Using to the hospital unless we believe something need to be taken an action that time, yes, we will definitely do the need for it.

Speaker 1: Okay. So now doctor, from your experience and from your review, what obstacles might pharmacists face when it comes to practice PV and reporting? ADRs?

Speaker 2: We're

Speaker 1: Talking about the hospital pharmacist

Speaker 2: Here. Yes, number one is limited number of staff and time. So time is always the big obstacle. So we have limited time and we have unlimited tasks and if we give the people task more than that, they cannot do it and it'll keep accumulation. This is obstacle number one. So there is no specific person assigned to do the A DR follow-up. This is number one. Number two, maybe the lack of the training about ADR reporting. Maybe we need more training for staff who will work on this area to be more specialized and to differentiate between side effect and ADR, et cetera. And also to be formalized with the system. Number three, absence of electronic system can be an obstacle to practice ADR reporting. Still now in our hospital we are manually filling the ADRs happened using ADR-reporting form. But we are planning to be electronic from next year. This again obstacle because if you have half manual have online still you cannot capture everything and especially if your system is not friendly used, so you cannot find easily the progress note, you cannot find the flag of there's a DR or et cetera. This is also an obstacle. That's it.

Speaker 1: And at the organizational level, something had to be done from the level of management at the hospital, do you think is there any barriers at this level?

Speaker 2: Generally speaking? Generally speaking and what I learned from antimicrobial stewardship leadership support is the essential for success for each and every task within the organization. So if there is leadership support, if there is commitment from the management of the safety of the patient, if they think safety is the first priority for their patient, definitely this will support that one. In our organization we decided to open, for example Saturday we have limited pharmacist, then I said we cannot do Saturday because we cannot do two people. Then when the CEO he said safety is first, although he want to start Saturday from next week. Then when he listened to us and we told this when co-signing is needed, then he said okay, I will postpone it till you have one more pharmacist. Then it was postponed for three months and once we have the pharmacist, then we start Saturday. So this reflect the commitment for the safety. So if there's any initiative related to safety, I believe it'll be taken from top down in the organization.

Speaker 1: So from your review, what changes in policy or regulation could help address and remove barriers to a DR reporting?

Speaker 2: Another problem I did not mention is the space. We have limited space so maybe we need office specified for the person who will be working for pharmacovigilance. He will be in his office with the resources to support him, different resources, references, internet, computer, access to the literature, all this. If we have and person specialized to do that, who will trained in this area, specialized in this area, definitely will be moving the pharmacovigilance from one level to second level and higher level.

Speaker 1: So how might a multi-stakeholder approach involving healthcare providers from pharmacists, nurses, the physician, regular bodies and pharmaceutical companies could improve the overall A DR approaching process from your review?

Speaker 2: Again, with the taking example of antimicrobial stewardship program because this is my area of interest, but really if I can adapt the same thing to any program, it'll be successful. So there is a core element to make the stewardship successful here I can say also core element to make a DR successful. So number one, commitment from the leadership. We already highlight this point. Then a multidisciplinary team who look to that one, but there should be a single person responsible for this program maybe with co-share, maybe physician plus pharmacist to be the core member and the co-leader for that program. Then other people like IT, authorities, nurse, PTC representative, all this is as a member to help in establishing this and then reporting. We need to report all that with the KBI. So number of number a DR reported number of missed A DR not reported.

Speaker 2: So we need to see and we need to reach to a target where a hundred patients should be reported. But the question how to capture unreported a DR, this is the real problem. How to capture and after reporting how to make everybody reporting, maybe we need to make an incentive for the people who report more so the top reporter for ADR in the organization. Maybe we can give him certificate in quality day that he's the highest number of reporting ADR or who prevent more ADR of that etc. And then cost. What's the cost associated with this program? And there should be maybe budget assigned for such work. Because really anything need work, it needs time and if it needs time, it need people and if it needs people it need money at the end of the day.

Speaker 1: Alright, so what kind of studies are we going to need in the future to improve the reporting of A DR by UA healthcare providers in general? As you have an experience in research and academic, what kind of research do you think that can help improving a DR reporting?

Speaker 2: Maybe we need to find the real number of missed A DR. We need to do a study to look how many ADR and medication errors were not reported; I mean underreporting. So if we highlight this point and type of underreporting sometime maybe it's a killing error or ADR, but it's not reported at all. So we need to do study to look to this and categorize them and to pharmacoeconomic study to calculate the amount of money missed or lost because of unreported idea. This will really make people think how much we are losing because of ADR.

Speaker 1: Nice. So at the end again, the last question. To which extent do you think conducting continuous education sessions and training for pharmacists about a DR reporting the process and BV is important or it can help to improve a DR reporting?

Speaker 2: Definitely training is always a core one of the element of success for any program because okay, don't let the people do it as they learn, but give them exercise and give them training how to do it the right way from the beginning. So if it'll become easy for them, it'll be part of their daily task. But if you told them, okay, reported without the training, I think it'll be slow learning process. It'll be learned one day, but the time that will be taken is more than what you expected

Speaker 1: At the end. Thank you so much s for your valuable time and for all your feedbacks and comments and it was my pleasure to meet you. Thank you so much.

Speaker 2: Thank you very much and I hope good luck for you and for your research and my regards to your supervisor, co supervisor and all your team. And I hope you will publish your study as soon as possible. Thank you.

Speaker 1: Thank so much. Thank you.
