# Supplementary material for: A qualitative study to investigate pharmacovigilance systems in Dubai hospitals
Source: PLoS One. 2025 Sep 10;20(9):e0331929. doi: 10.1371/journal.pone.0331929 (PMC12422479; doi:10.1371/journal.pone.0331929)
Supplement: S2 File — (ZIP) [file pone.0331929.s002.zip › Dr Muhamed SGH-Dubai-Done.docx]

Speaker 1: Hello? Hello, Dr. Hammad. Good morning. How are you?

Speaker 2: Good Morning Dr. Sawsan. I'm good. How about you?

Speaker 1: Fine. Good Hamdellah. Thank you for accepting my invitation. If you don't mind, can you open the camera because it has to be video

Speaker 2: Actually I'm working in the pharmacy so I'll talk and I'll work outside.

Speaker 1: Okay, fine. Okay, so just I will start it quickly. I sent for you already the consent form.

Speaker 2: I’ll will share it with you

Speaker 1: Yes, thank you so much. So I will start here. I'll not take a lot of your time. Can you please first introduce yourself? What is your qualification, your ranking, your job title at the hospital and your years of experience, country of graduation?

Speaker 2: My name is Mohammad Madni. I, I'm Pharm D. Basically I'm graduated from Baze University, Muzan Pakistan and my current job title is inpatient pharmacy manager in Saudi German Hospital, Dubai. I'm working here since last 11 years and total I have like 15 years’ experience. Four years I worked in Han Hospital Karachi.

Speaker 1: Okay, excellent. So you worked there for four years, then you shifted to Dubai?

Speaker 2: Yes, exactly.

Speaker 1: Okay. So just I will go directly to the pharmacovigilance, I need to know more details about the system. In your hospital, is there any pharmacovigilance center or anybody assigned with the responsibility for monitoring ADR reporting in your working place?

Speaker 2: Yes, exactly. See we have a quality in-patient safety department, so all the medication errors and adverse drug reaction are going to be reported to the quality in-patient safety departments and then they are doing all the needful activities like why it has happened. Just they're doing all the root cause analysis and then they are intervening the concern person that how this error has been happening and how we can prevent this in the future. So this is how we are reporting our errors.

Speaker 1: Okay, so these are the quality department or they are severity pharmacovigilance center?

Speaker 2: You can say that our pharmacovigilance center is under quality. Quality patient safety department.

Speaker 1: Okay. And they are belong to the pharmacy or no?

Speaker 2: They are different healthcare professionals. They are doctors, they are nurses and pharmacists is also involved because personally speaking I am involved since last 11 years along with QPS for all these activities. So I'm the quality coordinator from pharmacy department.

Speaker 1: Okay, excellent. So does the pharmacovigilance center physically exist or no, everyone is working from its place?

Speaker 2: There is no dedicated center with the name of pharmacovigilance in our hospital. As I told you that our quality and patient safety department, they are all dealing with such kind of things.

Speaker 1: Okay, fine. So is there a clear meditate, organizational structure, rule responsibilities and reporting lines for the pharmacovigilance center or the people working in this one? Is this very clear in the hospital?

Speaker 2: Yeah, it's very clear from the management and from the top management and from the quality and patient safety department that all the medication errors and adverse drug reaction will be reported via adverse drug reaction reporting form. There is another mechanism called OVR, generation occurrence variance report generation and then after the generation of that OVR or adverse drug action reporting or any medication error reports, then the need for actions are going to be done after that.

Speaker 1: Okay. And I will ask more details about this point, the process, but first I need to know is there an annual budget allocated for the pharmacovigilance activities at your hospital?

Speaker 2: There is no specific budget for pharmacovigilance activities at the hospital. As I told you that QPS is dealing with all these things. You can say that they are hiring the staff in the QPS department and they are doing all this. So the staff who is dealing with this, there is one dedicated staff who is dealing with all such type of things even in QPS department also and from pharmacy side also. So you can say that the budget is there for those staff of course but For example if you will say that visibly they are allocating a dedicated budget with the name of pharmacovigilance activities. No it's not like that.

Speaker 1: So it is within the budget of the pharmacy or with the other department?

Speaker 2: Yes, Exactly. Because all pharmacy for example, our basic job is to review the prescription for appropriateness. Among this one of our activity is identifying or tracking the adverse drug reaction and reporting them and the medication errors in the prescription and then reporting them. So all the pharmacists, this is one of the job description in the pharmacist role. So this is very clear from the management and from the policy and from the job description also that we need to do all these activities.

Speaker 1: Okay. So Dr, what is your experience with the ADR reporting at your current working place or previous places? The one in the UAE

Speaker 2:  In UA E, what is happening for example, if you will talk in within the pharmacy? Mostly we are tracking drug adverse reaction by pressure of clinic.

Speaker 1: Hello? Hello?

Speaker 2: Hello? Can you Hear me?

Speaker 1: Yeah, the voice was lagging yes DR, now it is fine.

Speaker 2: How we are tracking them because you know that we are not directly dealing with the patient most of the time. So in the pharmacy we are tracking adverse drug reaction by clinical trace drugs

Speaker 1: What Is the meaning of clinical trace drugs?

Speaker 2: For example, nurse is rushing to the pharmacy and she is asking for a stat dose of hydrocortisone and chlorochlor in mainly it means that there may be a possibility that there is allergic reaction from any medicine.

Speaker 1: So

Speaker 2: We are inquiring the nurse to check if there is any allergy or hypersensitivity reaction And she has confirmed. So after that we are reporting the ADR form. Okay, from this medicine this reaction has happened on the best of clinical test drugs. Number two, I will give you another example. For example, I received a certain dose of vitamin K and patient was taking warfarin, definitely warfarin side effect is bleeding or something like that. So certain dose of vitamin K, it is telling us that maybe INR is high. Now patient is experienced some adverse effects. Another example I can give you nelaxone is the antidote for opioid. So we are receiving the doses of nelaxone and patient was on PET or something like that. Then from these clinical test drugs in the pharmacy mostly we are tracking and identifying the adverse drug reaction and then reporting. Can you hear me?

Speaker 1: Yeah.

Speaker 2: Then after that from the nurses, because nurses are giving in the hospital 99% medicines to the patients. So whenever they are experiencing any adverse reaction, mostly they're reporting allergic reactions or hypersensitivity reaction. Mostly in our hospital. So mostly we are receiving from emergency department from ICU department from general ward or surgery department, something like that. Yes, they are well aware about our mechanism when they are facing any such kind of situation. They're filling the adverse direction reporting form and then they are submitting to the pharmacy. Actually I'm responsible for all these activities and what we are doing then we are making a list of all drugs and all patient name drugs, name, what are the causative events, other concomitant drugs and then this data we are sharing in the pharmacy and therapeutic committee for further action in order to know the root causes. Is there any related to increased or fast rate of administration of the medicine?

Speaker 2: For example, yesterday I received one ADR from fair inject injection. So fair inject is iron injection and with iron injection allergic or hypersensitivity reactions are common. So once I received the A DR, what I did, I called the nurse that at which rate you have administered this medicine because if you will administer iron with fast rate of administration what will happen due to intolerability hypersensitivity or allergic reactions will occur. But if you'll administer it slowly so these things will not come. So in order to identify a hundred percent correctly whether the patient is really allergic to iron or that specific product or it is due to fast rate of administration of the medicine. So after inquiring from him she told that okay, I gave at the start one ml per minute or something like that R 20 or 10 drops per minute. So from this we have extracted that no this is not related to infusion rate. This is purely the patient is allergic to the product. So this is how we are doing in our hospital.

Speaker 1: So after they report it for you where it'll go after that

Speaker 2: Yeah they have reported to me and then as I told you that we have one report, Excel report we're preparing in order to be shared in the pharmacy and therapeutic meeting to take further investigation and they are generating OVR also to QPS department because QPS department is also investigating that why this adverse reaction has happened. You know that because of some adverse reaction are occurring due to idiosyncratic reasons we don't know the reason but patient is allergic to the med. But if we didn't find any root cause then we are documenting this in the patient record for future perspective because if it is not documented and again patient will come and the same medicine will be given to the patient of course it's not good. So we are sending an official email to the medical record department, please update this in the record of the system that this patient is allergic or this patient had experienced this kind of adverse reaction from this medicine. So that when doctor will open the profile of the patient next time when patient will visit the doctor, every doctor must know that this has happened with this patient.

Speaker 1: Okay, excellent. And do you have a system connected to the DHA, you are doing this reporting of ADR on a regular basis for them?

Speaker 2: it's not connected directly to DHA or MOH. Our QPS department, our quality and patient safety department is sending data to DHA about medication errors and serious adverse regulation happened

Speaker 1: Yes, like every, how many they sending for them these report quarter for example?

Speaker 2: I think they're sending quarterly

Speaker 1: Quarter, sorry, quarterly.

Speaker 2: Yeah they're sending quarterly,

Speaker 1: Yes. Yeah. And as you said the most type of ADRs came from the ER department. The emergence? Yeah,

Speaker 2: Mostly. Mostly we are receiving the reports from the ER department most.

Speaker 1: And what are the most common types like the allergic reactions?

Speaker 2: Allergic or hypersensitivity mostly

Speaker 1: And you can consider them like a minor.

Speaker 2: It depends on the severity because sometime the allergic reaction is severe so we cannot say it's mild to moderate. We are documenting a severity as severity.

Speaker 1: So you are reporting all types of ADR?

Speaker 2: Yes, we do report all types of ADRs regardless if they are major or minor, severe or mild. Rare or common. All ADRs and medication errors we do report them.

Speaker 1: Okay. doctor, what do you think the obstacles that might pharmacists face when it comes to practice PV and reporting ADRs?

Speaker 2: The biggest challenge to be honest with you is number one is availability of the staff. If staff is less, if staff is less and work is more, then people will not care to report. yeah, people didn't find that time and know when any OVR is going to be generated again then investigation mechanism is going to be started. So staff has to answer them a lot and unlike this, this is number one thing. Number two thing is that lack of awareness and training. For example, if one staff is coming from India or Pakistan and they're from the remote areas, they don't care about these things.

Speaker 2: So if somebody will not educate them properly that this is really a patient safety concern and if you face that kind of situation and if you will not report it to the pharmacy and patient quality department, patient and safety quality department, how we can develop the strategies to minimize them or to control them. For me as per my experience, these are two main things that the staff should be well aware and there should be a sufficient number of staff so that they should not be overloaded with the work and they should feel the responsibility of reporting these important things. These are two main things for me.

Speaker 1: Okay. And do you think there are some cultural organizational barriers that also that may prevent the effective reporting ADR like at the managerial level?

Speaker 2: To be honest with you, in our hospital nothing like that. They're supporting now. They're supporting, they're encouraging. Okay, please report because it's very important so that we can do something for the patient's safety. This is what is happening in our hospital, what I have seen.

Speaker 1: So from your view, what changes in policy or regulations could help removing barriers?

Speaker 2: Maybe first thing we need, is to establish PV center in each hospital with a pharmacist specialized for this job. Then train this person how to deal with ADR reporting system at the hospital level and maybe he will be the person assigned lateron to train the other staff.

Speaker 1: And how do you think technology and data management systems can contribute to remove these barriers and encourage a DR reporting the technology and the data management system?

Speaker 2: Of course there is a role of technology and data management system. If there are well developed e system in the hospitals and they'll report and some rewarding system should be there for the staff also. For example, if you will tell the staff, okay, let's pause for example, one staff is doing everyday work and if she has given for example 30 medicines to the patient in one day, definitely there will be at least one adverse reaction. If you're giving 30 medicine, definitely there will be one and she is not reporting because there is overload, there is no encouragement, there is no recognition for her. So how she'll report, so you can provoke the staff or persuade the staff. Okay, once you report, we will give you some certificate, we'll give you some recognition, we'll call you in the grant staff meeting. Of course people like to be recognized and to be appreciated and some for example if you will give him some gift for example, anything like that. I'm not saying from my side because recently a few months before we had an GC, A audit from America and they also recommend this thing that okay you should encourage the staff by giving them some gift, some shield, something like that. So of course this will promote also and decision support system and e systems, they're also very important. For example, one adverse drug reaction has happened in the general ward

Speaker 2: And if it is not documented in the e system, the patient is coming after three days in the emergency department how doctor will know that this adverse reaction has happened already with this patient. So once he is opening the patient profile and it is clearly mentioned that okay this patient is allergic to cpro fluxes and of course there is a role of technology definitely.

Speaker 1: Okay, excellent. So this was my next question, how we encourage the hospital pharmacist to report a DR, you said to give them an incentive recognition. What else do you think that also can improve this A DR reporting practice to make it like a habit for everyone, not only for the pharmacist, even all the healthcare providers in the hospital.

Speaker 2: As I told you, proper training.

Speaker 1: Training,

Speaker 2: Yes proper training, reward and recognition system and make them aware about the significance of reporting ADRs. If they'll not do then what can happen? I mean we have to give them some persuasion so that these are the main things.

Speaker 1: And do you think the patients also play a role in reporting ADR?

Speaker 2: Of course. Why not? Because patient is the one who is experiencing all these things. For example, if one patient is taking PPA for example, it's a chronic ulcer patient and it's taking PPA for many months and now patient is experiencing bone weaknesses because PPA long-term use causes osteoporosis. So if we'll create awareness and doctor will discuss, our pharmacist or nurse will discuss all these things with the patient that while taking your medicine, if you experience any untoward effect, so you should share with us and this not only will give us or will make us unable to do something for you because medicines are not like our nutritional parts. They are xenobiotics, they some chemicals coming from outside and ultimately our body has to excrete them. So definitely all medicines have some kind of adverse effects or as you can say side effects, not adverse effects, side effects. So sometimes because of genetic variability or physiological variability, biochemical variability, different patients have different responses to the same drug. So if we'll discuss all these things with the patient, definitely it'll matter and it'll enhance the safety also.

Speaker 1: Okay, excellent. So doctor, are you willing to conduct an educational module if I would like to conduct this one for your staff at the hospital, like the pharmacist mainly

Speaker 2: See we are planning one safety week, I'm not sure within this couple of days, maybe within next month because my h RT was discussing this thing with me yesterday also that we have to celebrate one awareness week and we will inform the nurses something like that so that there will be awareness as I told that there should be a campaign or there should be an educational activity about PV to be given for all physicians, nurses and pharmacists. I will discuss with my HOD and if he'll agree why not, we'll call you and okay we will do from our side also and your contribution will also be there and your work will be done also and our work will be done also. Why not?

Speaker 1: The last thing I would like to ask you, what kind of studies do you feel that we need in the future to improve reporting of ADR in the UAE from your Experience

Speaker 2: The main thing is mostly Reporting ADR. Mostly people don't practice. This is the reality, people don't care that what we have to do, especially even within the hospital setting. For that, maybe we need a study to calculate the cost we lose in case we don’t do reporting. Also if I will say now it's a general public, okay, general public sponsors are always like that. But if we are talking about any healthcare system, people are not encouraged to report all these things. People are not well aware about these things. They studied this thing in their coursework and after that they're following the everyday routine. So if we'll make them aware and some rewarding system because you know that rewarding system is very important. Not give any reward even I have listened in my hospital why we'll do this thing. This is an extra burden or extra work for us. So there should be something like that so that people will practice.

Speaker 2: People have to feel good to report all these things. And then ultimately the benefit is for the whole population, for the whole community. Not only for that person,

Speaker 1: Do the physician do reporting or depends on the nurses to do the reporting?

Speaker 2: Physicians are reporting rarely. Rarely. If they want to report they're the best people to report also because patients is coming to them and patients are sharing their complaints to them also. So nurses and the physician do rarely.

Speaker 1: What about pharmacists?

Speaker 2: Pharmacist as I told you, outpatient. Outpatient, they can only do.

Speaker 1: Sorry

Speaker 2: Outpatients have to be honest with you. They are outpatients, they're bombarded with the work if patient is in front of them and then work is more and staff is less they can do if they want. It depends on their willingness also and how we can enhance their willingness. This is another subject. Yes. Why not every pharmacist healthcare practitioner, they can report who is in patients himself or herself also.

Speaker 1: So the last question, do you think that the ADR is part of the responsibilities of the pharmacist or no?

Speaker 2: Of course, of course. Why not? Because for example, our basic job as a pharmacist in the hospital is to do appropriateness review for the medication orders and then after doing the appropriateness review for the medication, we have to dispense the medicine. This is the basic job of the pharmacist, whether community, outpatient, inpatient, so in the appropriateness review we have to make sure that this drug is appropriate for that and one of the aspect is adverse drug reaction. For example, if I'm not checking the allergy

Speaker 2: And patient is allergic to super fluxes and or many times we have a practice doctor is writing allergic to penicillin in the record and in the active al we are seeing doctor is writing augmented tablet and Augmentin is amoxicillin, which is penicillin also. So because of you cannot say it is carelessness, sometimes it's not coming in the mind because doctor is overloaded and we have many other things, not only the profession. Sometime because of these things they are forgetting. For example, many times we have seen allergic to penicillin and doctors given piperacillin and taum because in the mind of the doctor, the brand name is Tazocin and he don't know that Tazo contains piperacillin and taaz. So sometime it happens. By the way, very strange things are happening, which we have seen in our practice. So yes, we have to do awareness session, we have to remind them again and again and we have to share a significant data with them so that after seeing the data, yes this thing is very important and we have to play our role also.

Speaker 1: Thank you so much doctor. Mohammad would like to add anything at the end related to ADR reporting?

Speaker 2: No, I don't want to add anything. This is from my side what I have seen in my practice.

Speaker 1: Okay. Thank you so much for your time and for your Thank you so much. Have a nice day.

Speaker 2: Thank.
