# Supplementary material for: A qualitative study to investigate pharmacovigilance systems in Dubai hospitals
Source: PLoS One. 2025 Sep 10;20(9):e0331929. doi: 10.1371/journal.pone.0331929 (PMC12422479; doi:10.1371/journal.pone.0331929)
Supplement: S2 File — (ZIP) [file pone.0331929.s002.zip › Dr Shadakasharya NMC DIP-Done.docx]

Speaker 1: Good morning.

Speaker 2: Good morning madam.

Speaker 1: How are you doctor?

Speaker 2: Fine, fine, fine.

Speaker 1: How is everything? Thank you for accepting my invitation and thank you also for signing the consent form. I already received it and just to let you know, this will be recorded video and voice and it can be used for publication later on, so just to take your approval.

Speaker 2: Yeah, it is fine

Speaker 1: Thank you so much. Sorry if my voice is not clear because I was sick the last few days.

Speaker 2: Yeah.

Speaker 1: Okay, so first can you please tell me how I will announce your name correctly?

Speaker 2: You can call me Shaak.

Speaker 1: Okay, fine.

Speaker 2: Short name. You can call me Sada

Speaker 1: Ada. Okay, Dr. Thank you so much. Okay, so first of all, can you please introduce yourself, your qualification, your ranking, the country of graduation and your years of experience?

Speaker 2: Yeah, precisely. I am the pharmacy graduate in 2002. In this field I am since almost 16 years and in the private hospital I worked for six years. Then the private retail pharmacy, I was with Taman industrial area in the retail pharmacy. Okay. Then I moved to hospital section. It is the third hospital. Last 12 years I was in the hospital and what to say, I was with Julia Hospital Dubai for three years and for five years in shaikh Kalifa Hospital since 2018 and last one year I promoted to IP pharmacy in charge and proud moment is actually I got three American board certification in pharmacy. One is with the company preparation in 2020 and 20 21, 20 22 with 2021 with CP hq and last month I got it pharmacotherapy, DPS score.

Speaker 1: Excellent, good. And now you are currently

Speaker 2: As an IP, inpatient pharmacy incharge pharmacist in NMC Royal Hospital, DIP.

Speaker 1: Okay.

Speaker 2: Dubai.

Speaker 1: And you got your bachelor degree from where?

Speaker 2: From Raju, Gandhi, university India, Bangalore.

Speaker 1: Okay, excellent. And you practice there in India or you come directly here to UAE?

Speaker 2: I practiced in India one year, then after then I moved to Dubai.

Speaker 1: Okay. Long experience. So now I will start about the pharmacovigilance topic. So if I need to ask you, is there any pharmacovigilance center or anybody assigned with the responsibility for monitoring ADR reporting in your working place?

Speaker 2: In the current hospital we have actually me as a pharmacy manager to be responsible. I am the one who reporting as an quality performance indicator of this one. As other pharmacist in the pharmacy have other tasks and they don’t know how to fill the report of ADR. We included this pharmacovigilance for example, we have medication errors, we have and adverse regulations we have

Speaker 1: is the pharmacovigilance center physically exist or no, it's just you are monitoring from your desk?

Speaker 2: We are monitoring, no center, we have software also.

Speaker 1: Software system where they can raise the ADRs? Yeah. Reactions immediately and all the things will go through the root cause analysis and it'll be justified. What is the reason and the corrective action will be taken? We have a software called a Sapphire.

Speaker 2: Yeah. Reactions will be taken immediately and all the things will go through the root cause analysis and it'll be justified. What is the reason and the corrective action will be taken? We have a software called a Sapphire.

Speaker 1: What is the reason and the corrective action will be taken?

Speaker 2: We have a software called a Sapphire.

Speaker 1: Okay. Later I will ask you in details about this software where and the process exactly in your working place. So now currently you are the one who's responsible about the pharmacist and ADR reporting?

Speaker 2: Yes. The reason being actually we are accredited by the JCI third time.

Speaker 1: How many pharmacist you're in the pharmacy

Speaker 2: In the JCI? This is a essential parameter actually.

Speaker 1: Okay. Essential parameter, okay.

Speaker 2: Yes, yes, yes. Yeah. That is going to be measured. There will be two types of measuring element. One important point is measuring elements. So and medication errors are measuring elements in the JC accreditation.

Speaker 1: Okay.

Speaker 2: In the inpatient pharmacy we have 12 pharmacist,

Speaker 1: 12 pharmacists. Okay. And for the outpatient

Speaker 2: We have around 30 pharmacist

Speaker 1: 30 excellent. Three zero. Yes. 30 pharmacy. One, three. Okay. So is there a clear meditate organizational structure rules related to ADR reporting in your working place? Like it's a mandatory,

Speaker 2: That is a mandatory and very much should because we are rely on only on the software updates in the computer system. We use the software called H-I-S-H-I-S means hospital information system.

Speaker 1: Okay, excellent.

Speaker 2: Okay. Everything depends on, let's say it's information for example of the review we use for the medication dispensing, the first essential stuff, one of the essential stuff is allergy column.

Speaker 1: Okay.

Speaker 2: And allergy column will be monitored and regularly we checked for the before dispensing, before administration and for everything for the upper, from the pharmacist side, from the doctor's side, from the before prescribing. Also it is from the HAS system only.

Speaker 1: And who's usually do the reporting like more pharmacists, nurses, physician.

Speaker 2: There are two types we can say in this section. Adverse reactions usually happens with a test doses and administration area and this will be managed by the nurses and doctors.

Speaker 1: Okay.

Speaker 2: And prescribing usually there is no adverse reactions, nothing is there. This prescribing and dispensing and dispensing is by the pharmacist. They will catch up the medication errors through the test.

Speaker 1: Okay. And do you have a clinical pharmacist who are doing the round in the wards and they can check if there's any wrong in the dose or ADR is giving for the patients?

Speaker 2: No, we don't have any clinical service for that. We have the rounds actually but not as in for the ADR we have general clinical rounds, not specific specifically for ADRs only.

Speaker 1: Okay. And do you have pharmacists who are doing the round with the other healthcare provider teams?

Speaker 2: Yes. Yes. Complete case profile, they will go and completely direct drug interactions. Everything they will check, they will not specifically bind to a D but they will bind to one thing in the clinical rounds. Usually what happens in the headset once the ADR form, we have two systems. One they have to fill the form physically and the next method is they have to update in that health. Sometimes this will be taking time so our clinical pharmacist round in the make sure that has to be updated very quickly. The reason being for example, one patient is there, imagine one patient got the allergy in the operation theater for one medication if it is didn't updated, if it is a day case, same category of the medication can be prescribed as a discharge medication. So the discharge medications rely allergic column only on the HIS never been on the physical form. So that's the reason we always stress on that gap to fill it and we are filling successfully.

Speaker 1: And the pharmacist who's the one who's doing this to fill this down?

Speaker 2: Yes, immediately. Okay,

Speaker 1: Excellent. So is there an annual budget allocated for the pharmacist activities in your working place?

Speaker 2: No. No, there is no separate budget for ADR reporting activites because what is the reason? We will tell you what is, we have JCI KPIs 90% of the even DHA and MOH KPIs also and 90% of them are our corporate KPIs also and our hospital KPIs also. So this is an essential part of our routine monitoring. So it is a responsibility of the inpatient pharmacist in charge to take care of all these things

Speaker 1: And all with the pharmacy budget?

Speaker 2: Yes, yes. It is included already in his job responsibility so there is no separate budget for it.

Speaker 1: Okay. So now please doctor, can you share with us your experience with the ADR reporting either at your current working place or previous working place?

Speaker 2: Actually, this ADR very serious. Honestly, we can say there are consequences in the general terms.

Speaker 2: We can say it'll be in the preparations. We can say the damage it does in the log around the way in the monetary, in the economic terms we can use the monetary values for example, how much damage it can do to a patient or to a hospital or for anybody. We say use the logarithmic queue. Logarithmic queue means we can say it'll multiples like 0, 0, 0 1, 1000 thousand, 10,000 like this. It is not like 1, 2, 4, it'll not multiply. It goes in that log way. So most of the hospitals and most of the people who are in the quality team are in the interest team, especially with the drug reactions and drug adverse reactions. It is a very serious and best part of this one is it can be preventable. There are some things cannot prevent, this can be preventable just by careful observations. So we usually what to do, we have not only when I was with Sheik Khalifa, they had also had the excellent reporting system.

Speaker 1: Okay you mean ADR reporting system?

Speaker 2: Yeah, they used to call actually Q program.

Speaker 2: Q program in the queue program we used to whatever the even medication errors everybody can put immediately in the system and automatically it'll go to the quality team. Quality team will address to the concern department to the root cause analysis. And what are the precautions to be taken? When I, this was between 2000 to 2018 with the she kfa around 2010, 11 in total I was with jica. Okay. No Dubai. Dubai, excellent. They also similarly they also had the one software, they didn't have any reporting software system but everything was the manual during that moment they used to fill the form, they were serious with the ideas and once it is filled the form and pharmacist we will go and make sure files are updated with allergic column and one more different system they had because JU Hospital had a software with built by own people so they had a coding system in their trucks. For example, AM Moline have only one code.

Speaker 2: All amoxicillin will, it'll used to identify there is a different in the coding system itself they identified there were the four parts of the coding systems. The first part will address that one as a generic name. For example, amoxicillin. Amoxicillin have one code. If the adopter enter, if it is a system is updated amoxicillin, all amoxicillin brands will be prohibited for the prescribing, dispensing and administration for that particular patient. Okay. They use in the information technology we can say in the pharmacy informatics language we can say this is called clinical decision support system CDSS. You can write down this point, this is very amazing clinical decision support system means once it is identified and it can be used for the clinical justification itself. After then I work with the she this doesn't have this facility and then I worked working with NMC still this software facility is not there. It was there for example, I told you for example patient ea, the patient X is allergic to amoxicillin, all sort of amoxicillin generations products like amox and clade acid for example, Amox plain full range of either whatever the range strength 1 56 syrup, 2 28 syrup 3 75 tablet, 6 25 tablet, one gram tablet, two 50 syrup, everything full range. It used to be blocked. It used to not allow to prescribe itself.

Speaker 2: That is one of the best my experience. It says even though they have the reporting system as an manual might be they improved long because my head point was around 2012 almost now 10 years now maybe they would've already approved that system during that moment only they have that system after then later in the, it was not there but it was manual. It used to pop up but it used to allow.

Speaker 1: Okay so it gives like

Speaker 2: Yeah also popup only, but it'll allow you have to identify it.

Speaker 1: Excellent. And from your experience, what are the most common type of ADRs used to be reported? Like serious ADR, minor or all types of ADR?

Speaker 2: No, no, not all types are very rarely major. Actually most of them are, we can say skin allergic reactions only. Usually they use to identify very quickly. That's the main in this one and that is usually easily identifiable and they used to do very well. Nurses are very familiar with those things because they're trained for that one and they're doing very well.

Speaker 1: Okay, okay. Excellent.

Speaker 2: Rarely others are there very, it'll go to the TE where less in the respiratory breathing count and even some patients will go for hypotensive. Usually hypertensive cases will be reported from the operation data intersection where they have the excellent monitoring systems and excellent people are there around the team. Maybe outside it'll be minor and it's not easily identifiable if by the time it is identifiable already patient got recovered.

Speaker 1: So now Dr. At your working place, can you please provide us with the detailed explanation of ADR reporting system procedure at NMC? Where are you working now? How it goes exactly In details.

Speaker 2: Exactly In details. It's very simple. We have the reporting system manual and updating system in the HIS. We already provided all the areas A DR filling forms and it is in the A DR filling forms and it'll be oriented by me as a part of the medication management. MMU, there is one chapter from the A CI. This is an essential and a D will be, we'll put the form and we'll train each and every employee, especially the healthcare section, nurses and doctors and all the lab people, the radiology people, everybody except the non-technical people. What

Speaker 1: About the pharmacist also?

Speaker 2: Pharmacist. Usually we don't, this a DS comes under the monitoring system. We are not involved in the monitoring system of the patients rarely.

Speaker 1: For example, if patient came, outpatient came and he was complaining of any adverse drug reaction happened to him, allergic reaction or anything, any type or medication error and he informed the pharmacist. So what will be the rule of the pharmacist here?

Speaker 2: Yeah, pharmacist here. We'll not justify it clinically, it's an idea what we'll do in this case, we will send that patient to the consent department. If concern doctor is not there, we'll send that one to the ER.

Speaker 1: Okay.

Speaker 2: It may be something else that may be, for example, it is the extended reaction of the, we can say side effect. For example, any patient or any baby, it takes the amoxicillin for the first time in the life they'll get lose diarrhea or one or two times. It's a naturally expected side effect. It's not an adverse reaction and we'll send that one for the referral to the doctors here, doctor or department, they will clinically justify whether it is an EADR or not a DR because for example if you justify without clinical knowledge the whole life patient will be denied with that medication.

Speaker 1: Okay. So just to make it clear, the pharmacist here doesn't have any rule in ADR reporting at your working place?

Speaker 2: No. Medication error. Medication error we have but ADR, no.

Speaker 1: Okay. And how would go in the reporting?

Speaker 2: Once the report is filled, it'll come to me then I'll go through it. We have one scale to use

Speaker 1: Yeah, scale. Excellent. So, you are using assessment scale to check the ADRs?

Speaker 2: Assessment to check whether it's a true ADR or it's a false idea.

Speaker 1: And you are the one who's doing this assessment scan?

Speaker 2: Yes, yes, yes, yes. Yeah, we'll go through that one. We'll go through the side effects, we'll go through the patient parameters and all the parameters will go through and we'll justify whether it is an ADR or not then we will allow the things update in that as an ADR.

Speaker 1: Okay, so just to make it clear now, for example starting, you said that the physician nurses and other healthcare provider except the pharmacist, they are doing ADR reporting. Is it a soft copy? Soft copy or hard copy? There is a form they fill it

Speaker 2: Hard copy, hard copy,

Speaker 1: Hard copy. So once they send hard copy, where it will go?

Speaker 2: It'll come to me only

Speaker 1: To you. So directly they are all sent this report to you?

Speaker 2: Yes. Immediately they'll scan and they'll send an email to CCT quality team and they were able to responsibility team. I'll go through that scale and we'll update the I system.

Speaker 1: Okay, so you are updating the system you put there the causality assessment? No,

Speaker 2: I'll not update. I'll inform the consultant to update it.

Speaker 1: Okay. Is here any role for the quality department, you have it in your hospital in this stage or no like the final report comes from you or from them?

Speaker 2: The initial report comes to me. I'll go through the root cause and I'll justify whether it is an ADR or not.

Speaker 1: Okay.

Speaker 2: And will give the feedback to the doctor and he will classify the new ADR or it is a side of it.

Speaker 1: Okay. Then after that

Speaker 2: Then it'll be updated in the H-I-S-H-I-S.

Speaker 1: And is there anyone who's monitoring this HIS like following up and updated?

Speaker 2: Yes, yes, yes. This is the part of the ADR monitoring system till complete we have to justify.

Speaker 1: Okay. And after that, is it connected to the Ministry of Health or DHA? Like now do you send this report to the Ministry of Health or only it at the level of your hospital?

Speaker 2: Oh no, no. We will have the reporting system to DHA, also we are sending to them.

Speaker 1: So all types of ADR you are sending to them or only specific types?

Speaker 2: All types.

Speaker 1: All types. Minor, major, major.

Speaker 2: Everything. Everything.

Speaker 1: Everything. And is there a time limit that you have to send the report with? Monthly. Monthly. So every month you're sending all

Speaker 2: Usually depends actually if it is a patient is going to be intensive care, we don't have anything major until now, but my previous hospital we had one incident where it hand to the Stevenson Johnson syndrome.

Speaker 1: You can also send for the pharmaceutical companies?

Speaker 2: Yeah, exactly. If it's going to be aggravated patient condition going to be deteriorated, we have to send immediately.

Speaker 1: Okay.

Speaker 2: In that case we'll immediately intimate them and we'll call them and we'll get the feedback. If it's required they'll come also.

Speaker 1: Okay, excellent. Okay. If we talk about the medication error, you said the pharmacist play a role in this one, the medication error, not like the others.

Speaker 2: No, no, no.

Speaker 1: So can you give us please an idea what is the role of the pharmacist here?

Speaker 2: Most of the medication errors are happening with the prescribing errors.

Speaker 1: Okay,

Speaker 2: So we have excellent one appropriateness of the review system and if we want I will show one parameter for you. I'll show one. You can screenshot this one. I'll show you what are the app minutes of the review before it used to be manual, this is an excellent one. One we'll not show any patient specifics but generally that column is completely for every patient, for every medication. That column needs to be validate in the system

Speaker 1: Ok.

Speaker 2: Then only after validation of that method we can give the medications, we can dispense the medications. Otherwise it's not possible to do

Speaker 1: So. It is a soft copy for them to be felt like the form of medication error.

Speaker 2: Yes. No, I'll tell you now first.

Speaker 1: Okay.

Speaker 2: To reverse the camera?

Speaker 1: Yes, fine.

Speaker 2: Ah, here it's there. Yeah. See this one.

Speaker 1: Okay,

Speaker 2: See this Castro pan tablet. If I need to describe the upper dose row and road, second one is therapeutic duplication. You can see

Speaker 1: Yes, yes, yes. I can read it. It's clear. Yeah.

Speaker 2: Allergies and sensitivity. If it is there, see then allergies and sensitivity disease means yes. Then I can describe what is the remark here patient is allergic to blah blah blah blah blah and I can write and it'll go to the system in the file for the evaluation. Okay,

Speaker 1: So this one is used by the pharmacist, outpatient

Speaker 2: Pharmacist for example. These are the medications.

Speaker 1: Okay.

Speaker 2: For each medication this is the popup.

Speaker 1: Ah, so they have to fill all of this before dispensing the medication.

Speaker 2: For example, See this one again,

Speaker 1: So for each medication in the description they have to see then finalize the verification.

Speaker 2: Ah,

Speaker 1: Okay. Okay, nice.

Speaker 2: Yeah. See if you do your work, if you don't document your work, you are not done here. We've done our work, we documented our work and we can give the comments. Also still my suggestion to my management is this needs to be set as an alert alarm.

Speaker 1: Yes. If What?

Speaker 2: If you study Well pharmacovigilance are the safety measures, the advanced information technology, what it says it should once it is known, once it's not allowed, this alarm needs to be generated immediately and this alarm needs to go to the consultant immediately and he had to justify and he had to come back.

Speaker 1: Okay,

Speaker 2: Excellent. In the advanced of the software system, this system is allowed. Now we reached it to this system. Next my request for the next year for the IT improvement, this was my request

Speaker 1: In, sorry.

Speaker 2: Now at the moment they have to go to the only approval is there then we'll document and we will talk to the consultant and we'll correct the orders and we'll use the new medicine.

Speaker 1: Okay. Here what happens?

Speaker 2: Medication error, maximum error are related to the dose and frequencies and weight-based dosing also because what is the reason we house multi-specialty hospital in the ER too much patients with a different background. If he's the pediatric, he cannot assess the cardiac case properly. If he's the cardiac, he cannot assess the pregnant, not pregnant pediatric patient properly. He might do, he might do, he will do but in that case he may not familiar with the exact doses of the medication Here pharmacist approval system is there. We are eliminating that risk by our approval. This is the reason we're getting the medication errors in this section.

Speaker 1: You mean Pharmacist will they identify?

Speaker 2: They'll identify the error and they'll get the corrected order and they will take the front order before the order. They'll take the big order before and it'll be monitored and it'll be the KPN and we'll send the goat to exactly what happened with the doctor and we'll give the updated required material for that study and we as an effort up that one last year we got for whole whole hospital we got to related the medications Lexicomp as a reference material to that? Yes.

Speaker 1: Okay, excellent. So you have this reference for the pharmacist to be used

Speaker 2: Not only pharmacists, all nurses, all doctors. We be, we had reference we're doing hundreds of reference. Are there? We want to be in the same reference everybody.

Speaker 1: Okay,

Speaker 2: So then that moment we went through the Lexi camp still a load along the Lexi camp also according to the clinical discretion sub. Hello? Yeah, you can listen.

Speaker 1: Okay. It was lagging. Okay now

Speaker 2: Yeah.

Speaker 1: So in case one of these criteria become yes with the pharmacist he found any of these medication error, what the steps? He will take it.

Speaker 2: That's what he go to the doctor, he will call the consultant,

Speaker 1: He'll contact directly the physician

Speaker 2: Directly, he'll contact the physician and He'll contact.

Speaker 1: So he'll not come back to you.

Speaker 2: Huh?

Speaker 1: He'll come back to you as you are the on charge pharmacist or directly He will contact the physician Directly?

Speaker 2: Pharmacist will contact the physician and he'll ask what is the exactly is it he was going to be, this is the reference and this is the dose or you want to go through this dose or my sense says this is the different dose. Okay. Then sometimes physician will say, this is the reason I'm giving this dose and you can continue. I know I have the reference from my side. I'll take care of patients. Sometimes that will also happen. Most of the times they will correct the dose.

Speaker 1: Okay, so nice Interaction.

Speaker 2: 95% of the cases it'll go according to the pharmacist.

Speaker 1: To the pharmacist. Okay. Excellent. Okay, so doctor now from your review, what obstacles do you see that my pharmacist face when it comes to practice BV and A DR reporting? If we say that the pharmacist, we want them to practice.

Speaker 2: Yes. First of all, what we see is actually many experiences after my board certification in the last five years.

Speaker 2: A pharmacist has to be better than urologist. The urologist better than cardiologist. With the cardiologist better than pediatrician. With a pediatrician. This is the expectation from the society. It is the expectation from the other healthcare workers. So we have to be familiar with the primary sources for knowledge and we have to update our knowledge regularly because it's a very worst and very new guidance. New products are coming, new safety measures are coming and by God, grace you selected pharmacy, you are pharmacist, you have the authority to do and you have that you are the only authorized person to do trusted by the society and authorities. So we have to play our role very well. How we can play only with the knowledge.

Speaker 1: With the knowledge. Okay.

Speaker 2: I feel one of the best sources that American board, board BPS board notification specialization, that is just a namesake, namesake certification. The main thing of that purpose is the material they give will go through the study that is amazing. It'll go through the guidelines better to go through the references, stay updated and give the full assist. After what I had done here, my system, I made all systems in one shared folder. I put all the reference material there, board material, everything there, never anything. Just refer and then call the doctor. It doesn't mean everything he's wrong because he is the ultimate care of the patient. He have to answer to them. Even the regulatory authority like A-D-H-A-M-O-H and dha ultimately patient is in his hand. Our part is only pharmacy field, only medicine field. He have to take care of full. So before answering referral, well read very well, be ready with the perfect materials, supporting documents before document to the doctor and be polite with the doctor. Don't authorize just when you talk to the doctor, please give the your view of safety 50. Not like blah blah blah blah blah. You are wrong. No doctor, this is the dose, this is the reference I got. Do you want to continue or do you want to change it? I felt this is a different like these.

Speaker 1: Yeah, so if we can the barriers we can mention first of all the level of education of the pharmacist. If he has a higher degree or no or if he has an extra

Speaker 2: Higher degree, plays an important even higher degree along with a higher degree did not limit them because knowledge is not limited to anybody. Anybody can read, anybody can interfere.

Speaker 1: Okay, so knowledge, knowledge, knowledge.

Speaker 2: Yes. That is

Speaker 1: Very important. The first barrier is the knowledge. The second one, the way of communication skills with the very

Speaker 2: Important. Yeah, once you burn an error you are not the king. Once you burn the error you're not the king and don't rule as a king you are the servant for the patient safety you have to fail as a patient safety warrior, you have to play like a warrior.

Speaker 1: Okay. What about other barriers? Do you see that?

Speaker 2: Other barriers is the software. Software should not allow to prescribe once it is allergic and we can say it is under the informatics section and the policy matic section is very lagging. Pharmacists are not enroll in the IT professional. They have to enroll in the IT professional and if they enroll and if they update their knowledge, if there is so many board certifications are there for the MA software also if they update and if they work in that field, like in best serving service software is there by the Cleveland clinic, Cerner is there from the Abu Dhabi government hospital, so many Cerner, those are all very expensive. Even what is there, the software need to be updated once it is allergy, it's not allowed to be prescribed.

Speaker 1: What about the time and the responsibilities? Do you see it can be like barriers for the to practice ADR or no

Speaker 2: Barrier is nothing. It is actually whenever, if you consider everything it is finally concerned as monetary measuring element in terms of money. That's it.

Speaker 1: Okay. So do you think also is there any barriers at the managerial level in any hospitals?

Speaker 2: Yes, exactly. Where the book stop is how much investment you need.

Speaker 1: So can you talk more about this one At the managerial level level

Speaker 2: Because of IT section requests a lot of money for this one and they're telling with this without investment also we are already beating a hundred percent of the target. So let us not investment so much money we'll see it in the next budget.

Speaker 1: Okay and you talk the

Speaker 2: Manager always profit and loss.

Speaker 1: So you think so it has to be starting from the manager Nigeria level to believe that the pharmacist can play a role in the A DR reportings.

Speaker 2: They know very well and most of the Middle East hospitals and the no hospital, not only any hospital, they are with the software because of the data management easy and everybody the data, once the software is there and it'll automatically quality will be there and quality always trust the quality and body management regarding medications, ADRs related to medications only 99% is and they will define only in the pharmacy feedback.

Speaker 1: Okay. So doctor, from your standard point, what do you think the changes that has been done in the regulation or the policy to remove these barriers in the A DR reporting and enhance the pharmaco visa resistance practice?

Speaker 2: From the regulatory point of view already it's satisfied. Actually we cannot say it's the lagging behind because it is mandatory to report that's and software update be done. It is already doing everywhere and only thing is actually software update made to be compulsory as a pop-up, they cannot do pop-ups should be once it is because for example, I told you one software is there where it is the drug is identified as an allergy and it should not be able to prescribe. That takes coding actually. But you have to identify the drugs as a code and that code identify properly because if it is misidentified with the wrong coding, we will deny that really important medication to that page if it goes with the wrong way. Another important thing is to force each hospital to establish PV center with staff dedicated for ADR reporting practice at the hospital level.

Speaker 1: Okay. So do you think continuous education and the training can enhance monitoring

Speaker 2: Regular?

Speaker 1: Sorry, your voice is lagging. It's silent doctor. Yeah. Again, can you repeat this? That the continuous education and this training can enhance the A DR reporting?

Speaker 2: Yeah.

Speaker 1: Okay.

Speaker 2: Yeah. Continue education and update of the knowledge related to types of ADR and medication errors, the process of reporting all can enhance the ADR practice. Many people honestly I believe when I took the many people introduced, they have the clinical pharmacist designation from the M-O-H-R-D-H-A and if I ask what is the best scale to monitor the D, they don't know exactly what you mean by scale. Forget about the a d scale name, that means lack of knowledge.

Speaker 1: Okay.

Speaker 2: Okay. Can we conclude? I have one meeting after two minutes.

Speaker 1: Yeah, yeah. So at the end, what kind of studies do you think we need in the future for a DR reporting and pharmac?

Speaker 2: For example, there is what to say. Everybody studied during their study where when we say comes under the what to say, I forgot the name of the subject of pharmacy. There's a regulatory issues, pharmac and one more one. Legislation.

Speaker 1: Pharmacy. Legislation.

Speaker 2: Legislation. Exactly. There they have to include, they have the chapter A, they have to read that chapter. Again, once it comes to the, they have to understand what is the consequences of the A DR.

Speaker 1: Okay.

Speaker 2: If any small where the error happens, we can prevent that error. That's the main point. And we can play very important role in the patient safety. We have to be serious with those areas and we can play that safety patients patient safety rules. If we don't play our responsibility, because society respects us as a pharmacist and we are the expert in our field for the regarding medications. We have to give our hundred percent of our knowledge, our effort, our ethics responsibilities to the patient safety.

Speaker 1: Okay. At the end, thank you so much, Victor, for your time and for your reviews and input in this qualitative study and wish you all the best. And recording.

Speaker 2: Can you ask recording? I'll tell you.

Speaker 1: I'll stop the record. Yeah. Okay. I stopped it.
