# Supplementary material for: A qualitative study to investigate pharmacovigilance systems in Dubai hospitals
Source: PLoS One. 2025 Sep 10;20(9):e0331929. doi: 10.1371/journal.pone.0331929 (PMC12422479; doi:10.1371/journal.pone.0331929)
Supplement: S2 File — (ZIP) [file pone.0331929.s002.zip › Suliman Al Habib Hospital-Done.docx]

Speaker 1: Okay. Hello Dr. Ahlam. How are you? Fine, thank you. Okay. Today I just will do an interview with you regarding the PV system in your hospital. Okay. Can you please introduce yourself?

Speaker 2: Hi, I'm Dr. Ahlam, the head of hospital pharmacy in Dr. Sleiman Habib. I've been working here for five years. Before that, I had worked in different hospitals and outpatient pharmacies in Dubai. And before that I worked in UK also for around four years as a pharmacy manager as well.

Speaker 1: Okay. So in total, how many years? No show of your experiences

Speaker 2: Around 13

Speaker 1: Years. 13 years? No. And your country of graduation?

Speaker 2: For bachelor degree it was from Jordan and master degree from Bright University in UK.

Speaker 1: Okay. Can you please us about your experience with the ADR reporting?

Speaker 2: Actually we have a full system for ADR reporting, but we are not receiving lots of adverse drug reactions. Reports. The policy here, we report to pharmacy like in the last couple of months we didn't receive any reports. Three months ago we received one. So actually still the reporting is weak

Speaker 1: From where you receive it? Inpatient.

Speaker 2: Inpatient, like here we have our system in, we should report everything about adverse drug reactions about to the pharmacy. So for example, in the postnatal department, if they suffered from any adverse drug reaction, they should report to pharmacy and then we investigate and then we put an action.

Speaker 1: Okay. So they reported for you. Is there any person in charge in the pharmacy or for you directly?

Speaker 2: No, in the pharmacy they send to me and I put the pharmacist for Z as in charge for all this. So he can look at them, report them, investigate and he can help me to put a proper actions.

Speaker 1: What types of ADRs are being reported?

Speaker 2: Only major and severe ADRs can be reported. No need to report the common ones.

Speaker 1: Okay. About the pharmacist. And is there any connection with the DHA to inform them?

Speaker 2: DHA? It's through quality. The reports actually is not coming to us directly. We have a system here in our computer, it's in engaged. One of the things in GATE system, it's called adverse drug reaction reporting. It's called incident reports. It's not only about adverse drug, it's reporting for many things. Like if there is fall, if is any incident adverse drug reaction, it's consider an incident in the hospital. So all these incidents when we need to report to quality department and then equality will start to divert to the right department. So with the quality receiving adverse reaction, they will send it to us. But it's not only this system, it's not only for adverse, it's all adverse drug events. For example, error near mass, adverse reaction, any event. And then the quality will segregate, adverse reaction will be sent to US medication error, the near miss will be sent to us.

Speaker 1: Okay. And after that

Speaker 2: We will take an action and we will write the action and then the quality also will look. So if it's serious, they will send to DHA quality department will contact DHA and they'll send to them.

Speaker 1: Okay, so you send it back to the quality and they contact

Speaker 2: Yes. Quality, they do the action. It depends on the severity and they have a criteria, DHA, when to report.

Speaker 1: Okay. So regarding the reporting, how many days then you have to report for them within four days? 24 hours one week

Speaker 2: To us?

Speaker 1: Yeah, to report it. To the DHA

Speaker 2: Report to the system, yeah. DHA, I'm not sure how often long

Speaker 1: For the DHA from the QC

Speaker 2: Department here, I think within 48 hours you should report to the quality.

Speaker 1: Okay. 48 hours. Okay,

Speaker 2: Fine. Maximum, I think it should be less than 48 hours, the maximum. But for DHA, I'm not sure about the rules.

Speaker 1: Okay. Did you take any training courses regarding to pharmacovigilance or ADR reporting?

Speaker 2: Yes, we always reading about it and when anybody is joining the hospital, they should have a training about PV system and ADR reporting. We give training also for new students called safety medication safety for all new joiners. As a part from the orientation program, it's called medication safety. This is our part we explain about narcotics and then medication safety. Within medication safety we focus on adverse drug reaction reporting. So we provided training, we just took a training when we started and then we read always about it by ourselves. And then we are the training providers for the hospital.

Speaker 1: You are the one who's doing Okay. So is that like a continuous or repeating courses for the pharmacist?

Speaker 2: Yes, with all the new joiners and also in the pharmacy. In any occasion, any event we do medication safety presentation, medication safety presentation, part of it. It includes the adverse drug reporting.

Speaker 1: Okay. And as we said, there is a protocol

Speaker 2: Or Yes, policy Report

Speaker 1: Okay. For the reporting. Okay. Is it obligatory to report that there is a rule here for the nurses? Yeah,

Speaker 2: It should of course, but problem we can't catch. It's not reported. You can, That's it.

Speaker 1: What about the outpatient?

Speaker 2: Outpatient? As for our outpatient pharmacy, they should report also. We don't receive from them. Okay. They report to the case also. They should report.

Speaker 1: Okay. And here you said there is a pharmacist who's doing the analysis?

Speaker 2: It should be the pharmacy manager, but I get the task for him so he can be focused and he's preparing reports to monitor it. And if it's the adverse drug reaction is complicated, we ask clinical pharmacists to investigate. So the investigation is teamwork, but there is pharmacists here. I give him the task of quality.

Speaker 1: Okay. And is there a format for this report or?

Speaker 2: Yes, it's an electronic format.

Speaker 1: Electronic format, Okay

Speaker 2: Before it was papers, then they stopped the papers and then they said it should be on system. It's called incident reports.

Speaker 1: Okay. Incidents reports. So how many reports of ADR, let's say last year you received

Speaker 2: Maybe six or seven.

Speaker 1: Okay, so the average is six to seven reports per year from inpatient. The one know we are talking inpatient and you told me what is the after report. Any other comments would you like to add from your side regarding the ADR reporting, pharmacovigilance, maybe we can increase this awareness.

Speaker 2: It's still, we need to continue nurses to remind because doctors are busy always. Just two days ago we did also small training for nurses. Nurses should take responsibility. Maybe head nurses should focus on that because doctors are busy and nurses, they may help them to.

Speaker 1: So you feel that the nurses are the person who should

Speaker 2: Because for us, we don't have contact with patients here, so we don't know if the patient there suffer.

Speaker 1: What about the clinical pharmacist when he do rounding in the wards? Are the patient do directly for him? Any reporting? No. No, because the patient, I think they are not talking with him.

Speaker 2: Sometimes he's talking with them, but they don't complain from him. We didn't have any case that they complained to him. But the nurses and the doctors, they know they should report and sometimes they tell you, one of the doctors told you because we solved it, we didn't report. They think it's an only rash if it's solved. So I told them it's solved, but we need to stop it to happen with another patient. So that's I think the mentality of the doctor, that it is done, finished, solved, why we need to report. Why we need to report it.

Speaker 1: The last thing I would like to ask you, what kind of studies do you feel that we need in the future to improve reporting of ADR in the UAE from your Experience

Speaker 2: The main thing is to find the reasons for underreporting in the country. Calculate the money we lost in case we are not reporting. How the technology could enhance PV activities in the country.

Speaker 1: Okay. Thank you so much for your timing and for everything. Thank.
